# Assessment of Symptom, Disability, and Financial Trajectories in Patients Hospitalized for COVID-19 at 6 Months

**DOI:** 10.1001/jamanetworkopen.2022.55795

**Published:** 2023-02-14

**Authors:** Andrew J. Admon, Theodore J. Iwashyna, Lee A. Kamphuis, Stephanie J. Gundel, Sarina K. Sahetya, Ithan D. Peltan, Steven Y. Chang, Jin H. Han, Kelly C. Vranas, Kirby P. Mayer, Aluko A. Hope, Sarah E. Jolley, Ellen Caldwell, Max L. Monahan, Katrina Hauschildt, Samuel M. Brown, Neil R. Aggarwal, B. Taylor Thompson, Catherine L. Hough

**Affiliations:** 1 Division of Pulmonary and Critical Care Medicine, Department of Internal Medicine, University of Michigan Medical School, Ann Arbor; 2 Department of Epidemiology, University of Michigan School of Public Health, Ann Arbor; 3 VA Center for Clinical Management Research, LTC Charles Kettles VA Medical Center, Ann Arbor, Michigan; 4 University of Michigan Institute for Healthcare Policy and Innovation, Ann Arbor; 5 Health Policy and Management, Bloomberg School of Public Health, Johns Hopkins University, Baltimore, Maryland; 6 Pulmonary and Critical Care Medicine, Department of Medicine, Johns Hopkins University, Baltimore, Maryland; 7 Department of Emergency Medicine, Harborview Medical Center, Seattle, Washington; 8 Division of Pulmonary and Critical Care Medicine, Department of Medicine, Johns Hopkins University School of Medicine, Baltimore, Maryland; 9 Division of Pulmonary and Critical Care Medicine, Department of Medicine, Intermountain Medical Center, Murray, Utah; 10 Division of Pulmonary and Critical Care Medicine, Department of Internal Medicine, University of Utah School of Medicine, Salt Lake City; 11 Ronald Reagan-UCLA Medical Center, Pulmonary & Critical Care Medicine, David Geffen School of Medicine at University of California, Los Angeles; 12 Department of Emergency Medicine, Vanderbilt University Medical Center, Nashville, Tennessee; 13 Geriatric Research, Education, and Clinical Center, VA Tennessee Valley Healthcare System, Nashville; 14 Division of Pulmonary and Critical Care Medicine, Oregon Health & Science University, Portland; 15 Center to Improve Veteran Involvement in Care, VA Portland Health Care System, Oregon Health and Science University, Portland, Oregon; 16 Department of Physical Therapy, College of Health Sciences, University of Kentucky, Lexington; 17 Division of Pulmonary and Critical Care Medicine, Department of Mundeledicine, Oregon Health and Science University School of Medicine, Portland; 18 Division of Pulmonary Sciences and Critical Care Medicine, University of Colorado, Denver; 19 Division of Pulmonary and Critical Care Medicine, Department of Internal Medicine, University of Washington, Seattle; 20 Center for Clinical Management Research, VA Ann Arbor Healthcare System, Ann Arbor, Michigan; 21 Division of Pulmonary and Critical Medicine, Massachusetts General Hospital, Boston, Massachusetts; 22 Division of Pulmonary and Critical Care Medicine, Department of Medicine, Oregon Health and Science University School of Medicine, Portland; 23 The National Heart, Lung, and Blood Institute Prevention and Early Treatment of Acute Lung Injury Network

## Abstract

**Importance:**

Individuals who survived COVID-19 often report persistent symptoms, disabilities, and financial consequences. However, national longitudinal estimates of symptom burden remain limited.

**Objective:**

To measure the incidence and changes over time in symptoms, disability, and financial status after COVID-19–related hospitalization.

**Design, Setting, and Participants:**

A national US multicenter prospective cohort study with 1-, 3-, and 6-month postdischarge visits was conducted at 44 sites participating in the National Heart, Lung, and Blood Institute Prevention and Early Treatment of Acute Lung Injury Network's Biology and Longitudinal Epidemiology: COVID-19 Observational (BLUE CORAL) study. Participants included hospitalized English- or Spanish-speaking adults without severe prehospitalization disabilities or cognitive impairment. Participants were enrolled between August 24, 2020, and July 20, 2021, with follow-up occurring through March 30, 2022.

**Exposure:**

Hospitalization for COVID-19 as identified with a positive SARS-CoV-2 molecular test.

**Main Outcomes and Measures:**

New or worsened cardiopulmonary symptoms, financial problems, functional impairments, perceived return to baseline health, and quality of life. Logistic regression was used to identify factors associated with new cardiopulmonary symptoms or financial problems at 6 months.

**Results:**

A total of 825 adults (444 [54.0%] were male, and 379 [46.0%] were female) met eligibility criteria and completed at least 1 follow-up survey. Median age was 56 (IQR, 43-66) years; 253 (30.7%) participants were Hispanic, 145 (17.6%) were non-Hispanic Black, and 360 (43.6%) were non-Hispanic White. Symptoms, disabilities, and financial problems remained highly prevalent among hospitalization survivors at month 6. Rates increased between months 1 and 6 for cardiopulmonary symptoms (from 67.3% to 75.4%; *P* = .001) and fatigue (from 40.7% to 50.8%; *P* < .001). Decreases were noted over the same interval for prevalent financial problems (from 66.1% to 56.4%; *P* < .001) and functional limitations (from 55.3% to 47.3%; *P* = .004). Participants not reporting problems at month 1 often reported new symptoms (60.0%), financial problems (23.7%), disabilities (23.8%), or fatigue (41.4%) at month 6.

**Conclusions and Relevance:**

The findings of this cohort study of people discharged after COVID-19 hospitalization suggest that recovery in symptoms, functional status, and fatigue was limited at 6 months, and some participants reported new problems 6 months after hospital discharge.

## Key Points

**Question:**

How do symptoms, disabilities, and financial problems evolve in the 6 months after COVID-19-related hospitalization?

**Findings:**

In this national US cohort study that included 825 adults discharged from 44 hospitals, 75.4% of COVID-19 survivors experienced cardiopulmonary problems at 6 months compared with 67.3% at month 1. Decreases were noted at 6 months in financial problems (from 66.1% to 56.4%) and functional limitations (55.3% to 47.3%).

**Meaning:**

The findings of this study suggest that symptoms, disabilities, and financial problems remain highly prevalent—with some new problems—in the 6 months after COVID-19 hospitalization.

## Teaser

This cohort study evaluates long-term physical and financial outcomes of individuals who were hospitalized with COVID-19.

## Introduction

For many individuals, the effects of COVID-19 persist after the acute phase and result in prolonged symptoms and disability.^1,2^ This has led to widespread efforts to characterize the epidemiologic characteristics of such post-COVID-19 sequelae.^3^ However, accurate data remain limited, hindering our ability to counsel patients, caregivers, and policy makers and to plan relevant recovery-focused clinical research.

Several specific issues remain. First, we lack accurate estimates of the incidence of many post-COVID-19 clinical, functional, and financial problems. Available reports have described the prevalence of some symptoms at follow-up but have generally not been designed to compare symptoms and other problems with prehospitalization baselines.^4,5,6^ Second, national studies using prospective enrollment of patients across diverse hospitals and regions are limited; increased geographic and temporal representation may reduce the analytical impact of regional health resource strain, surge conditions, and practice patterns on observed outcomes.^7,8^ Third, many early reports have been cross-sectional, providing little information on how symptoms and other sequelae evolve.^9,10,11^

In this article, we present the results of a prospective longitudinal study among adults hospitalized for COVID-19 in 44 hospitals across the US. By enrolling patients during hospitalization and conducting follow-up at 1, 3, and 6 months, we address the knowledge gaps noted above and provide critical data to help guide clinical care, public health, and scientific efforts.

## Methods

The Biology and Longitudinal Epidemiology: COVID-19 Observational (BLUE CORAL) study is a prospective cohort study conducted through the National Heart, Lung, and Blood Institute Prevention and Early Treatment of Acute Lung Injury (PETAL) Network and member sites (eAppendix in Supplement 1). BLUE CORAL enrolled 1369 patients with COVID-19 at 44 hospitals in the US. Herein, we report a preplanned analysis of 6-month follow-up data. This research was approved by the Vanderbilt University Institutional Review Board, which is the central institutional review board for the PETAL Network. Patients or their surrogates provided written informed consent for participation; financial compensation was provided. The report was developed according to the Strengthening the Reporting of Observational Studies in Epidemiology (STROBE) reporting guideline for cohort studies.

### Participants

We included patients hospitalized within 14 days of a positive molecular test for SARS-CoV-2 with fever and/or respiratory signs or symptoms compatible with COVID-19. Patients were enrolled within the first 72 hours of hospital admission and were excluded from BLUE CORAL if they had opted for comfort care or were incarcerated.

Survivors who spoke English or Spanish, were not homeless, and who were neither substantially disabled nor cognitively impaired at baseline were eligible for follow-up. We defined substantially disabled as being limited in 4 or more activities of daily living prior to hospitalization due to limited sensitivity of some of our questionnaires to detect changes resulting from COVID-19 in patients with greater baseline disability. We included patients who were able to provide consent for themselves or for whom a legally appointed representative reported no evidence of cognitive impairment, defined as 4 or more problems on the Alzheimer Dementia scale.^12^

### Data Collection

Participants were enrolled between August 24, 2020, and July 20, 2021, with follow-up occurring through March 30, 2022. Posthospital surveys were administered by trained interviewers in English or Spanish to patients or their proxies at 1, 3, and 6 months after enrollment. Patients were contacted by phone beginning 21 days after hospital discharge. The 1-month follow-up interview was completed at a median of 50 (IQR, 31-64) days, 3-month interview at 116.5 (IQR, 100-128) days, and 6-month interview at 193 (IQR, 165-212) days. Interviewers prioritized responses from patients but included proxy interviews with people in regular contact with the patient and knowledge of their health when necessary. We allowed for survey completion over multiple phone calls or by mail, and used best practices for data collection and cohort retention.^13,14^ Race and ethnicity was identified using a combination of patient or surrogate report and electronic medical record review. Study data were collected using electronic data capture tools hosted at the University of Michigan (REDCap).

### Survey Instruments

As previously reported, we assessed cardiopulmonary symptoms using the Airways Questionnaire 20, the Kansas City Cardiomyopathy Questionnaire, and the Seattle Angina Questionnaire.^15,16,17,18^ Respondents reporting symptoms at any time were asked to compare their symptom severity with 1 month before their initial hospitalization. We assessed fatigue using the Patient Health Questionnaire-9.^19^ Symptoms that were not present before hospitalization or specifically reported as increased in severity were counted as new or worsened.

Disability was assessed by self-report of limitations in activities of daily living (ADLs) or instrumental activities of daily living (IADLs).^20,21^ We asked specifically about health-related limitations in ADLs and IADLs and compared the number of limitations at each follow-up survey with limitations present before hospitalization as identified during the initial in-hospital survey. We also asked patients to describe their perceived recovery toward what they could do physically and mentally before their COVID-19 hospitalization on a 1- to 100-point scale, with 100 representing all the way back to what you could do before COVID.

We assessed financial problems using the World Health Organization Disability Assessment Schedule 2.0 question (“Since your COVID-19 hospitalization, how much has your health been a drain on the financial resources of you or your family?”) and questions regarding job changes, time off work, and insurance coverage developed with the Mi-COVID-19 study using qualitative interviews.^2,22^

Quality of life was measured using the European Quality of Life 5-dimension 5-level instrument.^23^ Individual responses were mapped onto a US-specific value set to arrive at a summary utility score and compared across time points. We also present responses stratified by dimension.^24^

### Statistical Analysis

Continuous variables are summarized with medians and IQRs. We tested for trends in symptoms across survey time points using Somers D, a rank-based test for trend that accounted for clustering among individuals.^25^ To see whether observed trends were influenced by loss to follow-up or baseline severity of illness, we evaluated symptoms separately among participants who (1) responded at all 3 times, (2) received mechanical ventilation, and (3) had 3 or more comorbidities at hospitalization. For symptoms, financial problems, and disabilities, we calculated the proportion and exact binomial CI of those initially problem-free who reported new problems at month 6. We constructed alluvial diagrams to visualize transitions across levels of disability, perceived recovery, and fatigue. We then used logistic regression to identify correlates of cardiopulmonary or financial problems at 6 months. All models included demographic characteristics (age in decades with a corresponding polynomial term, male sex at birth, race and ethnicity [Hispanic, non-Hispanic Black, non-Hispanic White, or other/unknown], and primary insurer [Medicare, other, or unknown]). Race and ethnicity was identified through patient or surrogate report and electronic medical record review; other included American Indian/Alaskan Native, Asian, Native Hawaiian/Pacific Islander, other/declined to report, and unknown. We next added the number of comorbidities (0, 1, 2, and ≥3) and immunocompromised status (HIV/AIDS, current chemotherapy, current leukemia, current lymphoma, bone marrow transplant, solid organ transplant, chronic oral corticosteroid at doses ≥20 mg/d, or other immunosuppressive medications for autoimmune disease) at hospital admission. Next, we added hospital length of stay (in quartiles), highest level of care (ward, intermediate care, or intensive care unit), maximal oxygen delivery device (none, conventional oxygen, high-flow nasal cannula, noninvasive positive pressure ventilation, or mechanical ventilation), and vasopressor use. In addition, we included cardiopulmonary problems at 1 month or financial problems at 1 month in each model for the same problems at 6 months. We present model discrimination using C statistics. Comorbidities were identified using methods developed by Charlson et al.^26^ Models used robust variance estimates to account for site-level clustering. We used case-wise deletion because missingness rates were small for all variables (eTable 1 in Supplement 1). All analyses were done using Stata, version 17.0 (StataCorp LLC) and R, version 4.1.2 (R Foundation for Statistical Computing) with the networkD3 package. Statistical significance was defined a priori at *P* < .05 and all hypothesis tests were 2-sided.

## Results

We enrolled a total of 1388 participants; of these, 1029 participants were eligible for follow-up at month 1, 1016 at month 3, and 1012 at month 6 (eFigures 1-3 in Supplement 1). Interview completion rates were 72.7% at month 1, 67.2% at month 3, and 69.1% at month 6, and 825 eligible participants (444 [54.0%] were male, and 379 [46.0%] were female) completed at least 1 follow-up interview; 163 (19.8%) completed 1 interview, 189 (22.9%) completed 2, and 472 (56.3%) completed all 3. Median age was 56 (IQR 43-66) years, 253 (30.7%) participants were of Hispanic ethnicity, 145 (17.6%) were non-Hispanic Black, 360 (43.6%) were non-Hispanic White, and 67 (8.1%) were other or unknown. Detailed demographic data are given in eTable 2 in Supplement 1. Those completing all 3 surveys were older than participants only completing the first survey (median age, 59 [IQR 47-68] vs 51 [IQR, 38-65] years). Otherwise, there were no significant differences between these groups across demographic characteristics, baseline health, or severity of illness (eTable 3 in Supplement 1).

### Cardiopulmonary Problems

The number of participants who reported new or worsened cardiopulmonary problems increased from 452 (67.3%) at month 1 to 482 (75.4%) at month 6 (*P* = .001) (Table 1). Among participants not reporting new problems at month 1, 60.0% (95% CI, 52.2%-67.4%) reported new problems at month 6. The most frequently reported problem at month 6 was chest trouble on exposure to odors or fumes (28.2%) followed by cough (27.2%). The proportion of participants with new or increased supplemental oxygen requirements decreased from 18.9% at month 1 to 11.5% at month 6 (*P* < .001), while the proportion with new positive airway pressure therapy increased from 5.0% to 12.7% (*P* < .001). There were slight differences in the proportions of respondents reporting symptoms at 6 months across initial level of care and baseline comorbidities, although these differences were not significantly different (eTable 4 in Supplement 1). Observed trends were similar among respondents who completed all 3 surveys (eTable 5 in Supplement 1) and respondents with 3 or more comorbidities (eTable 6 in Supplement 1). Trends among participants receiving mechanical ventilation are described in eTable 7 in Supplement 1. Correlates of new or worsened cardiopulmonary symptoms at month 6 are displayed in eTable 8 in Supplement 1. In our complete model, the presence of cardiorespiratory symptoms at month 1 was associated with greater odds of cardiopulmonary symptoms at month 6 (adjusted odds ratio, [aOR] 3.25; 95% CI, 2.01-5.27). Conventional oxygen (vs no supplemental oxygen) was also associated with greater symptoms at 6 months (aOR, 1.71; 95% CI, 1.10-2.66]). Male sex at birth was associated with fewer cardiopulmonary symptoms (aOR, 0.59; 95% CI, 0.37-0.95).

**Table 1.** New or Worsened Cardiopulmonary Problems Over Time Compared With Pre–COVID-19 Baselines

Variable	Survey time point, No. (%)^a^			*P* value^b^
	Month 1	Month 3	Month 6	
No.	670	623	638	
Symptoms				
Chest trouble with odors/fumes	90 (13.47)	168 (27.23)	179 (28.23)	<.001
Cough	136 (20.27)	122 (19.65)	173 (27.24)	.001
Swelling in feet, ankles, or legs	73 (10.94)	132 (21.50)	148 (23.60)	<.001
Chest trouble when upset	105 (15.70)	147 (23.75)	149 (23.46)	<.001
Rapid or irregular heartbeat	119 (17.92)	117 (19.18)	122 (19.40)	.41
Breathlessness with sleep	95 (14.20)	118 (19.09)	137 (21.57)	<.001
Chest pain, tightness, or angina	97 (14.54)	110 (17.77)	109 (17.38)	.10
Breathlessness				
When laughing	109 (16.27)	102 (16.50)	111 (17.56)	.47
When lying flat	82 (12.28)	83 (13.43)	83 (13.15)	.56
Chest trouble getting around the house	93 (13.90)	86 (13.92)	96 (15.07)	.49
Chest trouble leads to going home early	93 (13.98)	95 (15.37)	91 (14.33)	.83
Any symptom	349 (52.56)	377 (61.80)	403 (63.56)	<.001
PAP therapy and supplemental oxygen				
Using CPAP or another breathing machine during sleep	33 (4.97)	68 (10.91)	81 (12.74)	<.001
Using home oxygen	127 (18.93)	82 (13.20)	73 (11.50)	<.001
Any PAP therapy or supplemental oxygen	140 (20.96)	126 (20.29)	131 (20.60)	.83
Any cardiopulmonary problem	452 (67.26)	463 (74.20)	482 (75.43)	<.001

Abbreviations: CPAP, continuous positive airway pressure; PAP, positive airway pressure.

^a^ Patients did not answer questions if they were uncertain. Missingness rates are given in eTable 1 in Supplement 1.

^b^ *P* value calculating using Somers D accounting for clustering of observations by respondent.

### Financial Problems

The number of participants reporting any financial problem decreased from 66.1% at month 1 to 56.4% at month 6 (*P* < .001) (Table 2). By month 6, 34.8% of participants reported using up all or most of their savings, 20.4% had been unable to pay for necessities, and 16.3% had been contacted by a collection agency as a result of their COVID-19 hospitalization. Among participants not reporting financial problems at month 1, 23.7% (95% CI, 17.8%-30.4%) reported new financial problems at month 6. There were again slight differences in the proportions of respondents reporting financial problems at 6 months across initial levels of care and baseline comorbidities, although these proportions were not significantly different (eTable 4 in Supplement 1). Observed trends were similar among respondents completing all 3 surveys (eTable 9 in Supplement 1). A total of 30.7% of participants reported a moderate, severe, or extreme drain on their family’s financial resources at 6 months as a result of their COVID-19 hospitalization. Predictors of any financial problem at 6 months are described in eTable 9 in Supplement 1. Both Hispanic (aOR, 3.74; 95% CI, 2.24-6.23) and non-Hispanic Black (aOR, 2.63; 95% CI, 1.65-4.20) participants had greater odds of financial problems at 6 months compared with non-Hispanic White participants, as did participants with other or unknown race and ethnicity (aOR, 3.28; 95% CI, 1.32-8.17). Financial problems at 1 month were associated with greater odds of financial problems at 6 months (aOR, 8.03; 95% CI, 5.12-12.61) (eTable 10 in Supplement 1).

**Table 2.** Financial Problems at Each Time Point

Variable	Survey time point, No. (%)^a^			*P* value^b^
	Month 1	Month 3	Month 6	
No.	677	624	639	
At any time because of COVID-19 hospitalization, have you…				
Used up all or most of savings?	215 (31.80)	203 (33.12)	221 (34.80)	.14
Been unable to pay for necessities?	133 (19.67)	137 (22.10)	129 (20.35)	.69
Been contacted by a collection agency?	65 (9.62)	85 (13.80)	103 (16.30)	<.001
Skipped or delayed medical care because of cost?	52 (7.70)	64 (10.32)	59 (9.29)	.20
Taken less medications because of the cost?	42 (6.22)	48 (7.75)	42 (6.62)	.72
Declared bankruptcy?	7 (1.04)	8 (1.29)	9 (1.42)	.52
Since our last contact, have you…				
Had a loved one take time off work?	264 (39.23)	173 (27.86)	145 (22.69)	<.001
Had to change work?	96 (14.26)	94 (15.19)	75 (11.74)	.13
Been told that insurance would not cover therapy or rehab?	32 (4.77)	36 (5.87)	45 (7.08)	.05
Lost a job?	56 (8.30)	49 (7.85)	41 (6.41)	.14
Been told that insurance would not cover equipment?	45 (6.71)	32 (5.20)	32 (5.06)	.14
Prevalence of any financial problem	449 (66.13)	369 (59.04)	361 (56.41)	<.001

^a^ Patients did not answer questions if they were uncertain. Missingness rates are given in eTable 1 in Supplement 1.

^b^ *P* value calculating using Somers D accounting for clustering of observations by respondent.

### Disability and Impaired Health

**Figure 1.**
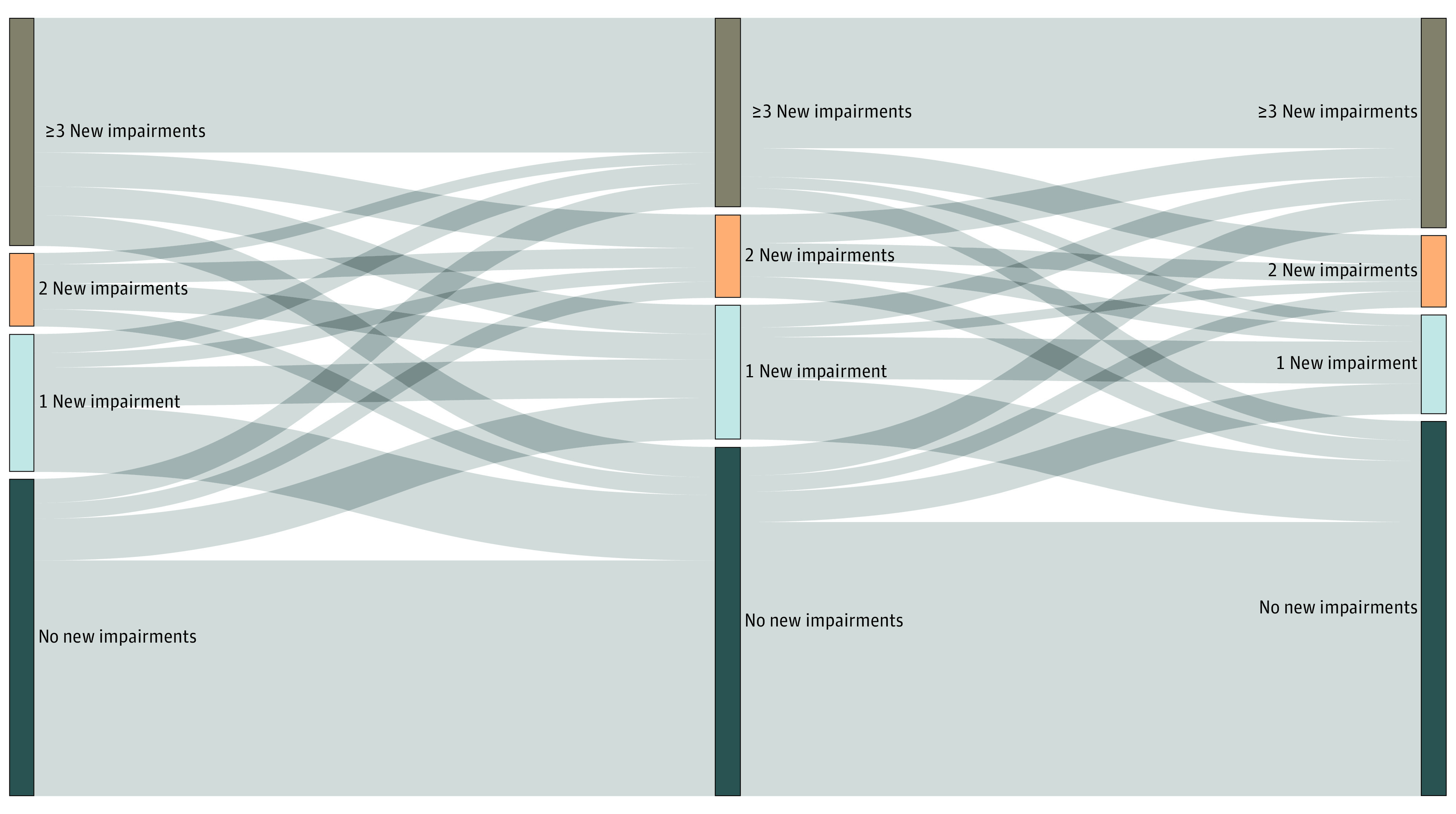
Trajectories in ADL and IADL Impairment Over Time Alluvial diagram displaying the proportion of respondents with activities of daily living (ADL) and instrumental activities of daily living (IADL) impairments at each time point (vertical bars) and changes in responses over time (horizontal lines). A total of 472 respondents provided ADL and IADL information during all 3 surveys. Bar and line thicknesses are proportional to the number of patients represented by the relevant category (vertical bar) or change (horizontal line).

Six months after hospitalization, 47.3% of participants reported at least 1 new ADL or IADL impairment, down from 55.3% at month 1 (*P* = .004), and 25.7% of participants reported 3 or more new ADL or IADL impairments at month 6. Among participants not reporting an ADL or IADL impairment at month 1, 23.8% (95% CI, 18.5%-29.8%) reported an impairment at month 6. Complete trajectories in ADL and IADL impairments are depicted in Figure 1. At 6 months, 9.7% of participants perceived their health as below 50% of their pre-COVID-19 baseline. Trajectories in perceived health among the 473 participants who completed all 3 interviews are depicted in eFigure 4 in Supplement 1. At 6 months, there were small differences in the proportions of respondents with new disabilities or who were below 100% of their baseline health across initial levels of care and baseline comorbidities, although these proportions were not significantly different (eTable 4 in Supplement 1).

### Fatigue

Six months after hospitalization, 50.8% of participants reported new or worsening fatigue, up from 40.7% at month 1 (*P* < .001), and 18.2% reported feeling tired nearly every day at month 6. A total of 66.3% of participants who reported fatigue at month 1 continued to experience fatigue at month 6. Among participants not reporting fatigue at month 1, 41.4% (95% CI, 35.7%-47.3%) reported fatigue by month 6. Complete trajectories are depicted in eFigure 5 in Supplement 1.

### Quality of Life

**Figure 2.**
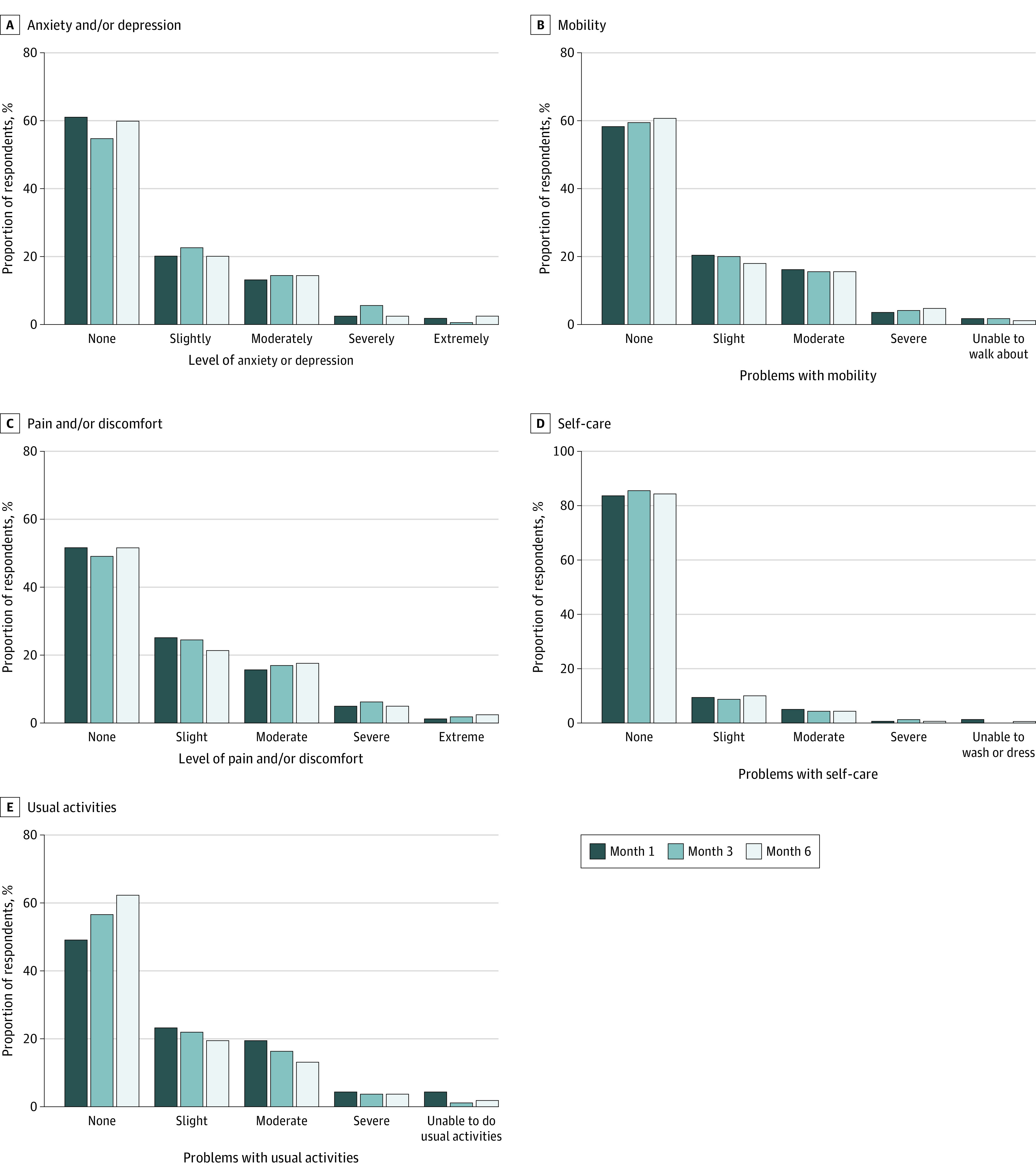
Self-reported Quality of Life Over Time, as Measured Using the European Quality of Life 5-Dimension Scale Responses stratified by time point and quality of life domain: anxiety and/or depression (A), mobility (B), pain and/or discomfort (C), self-care (D), and usual activities (E).

The median quality of life utility score remained stable between months 1 (0.85; IQR, 0.68-0.94), 3 (0.85; IQR, 0.66-1.00), and 6 (0.88; IQR, 0.66-1.00) (*P* = .04). These values were between those reported among participants with fair (median, 0.69) to good (median, 0.88) health in a US-based study establishing population norms.^27^ Dimension-specific quality of life responses are displayed in Figure 2.

## Discussion

In a diverse nationwide cohort, we found that cardiopulmonary, financial, and functional problems were highly prevalent 6 months after COVID-19 hospitalization. Some participants reported recovery during the study period; for most patients with problems at 1 month, however, improvements in symptoms, functional status, and fatigue were limited. Moreover, new problems often occurred later in follow-up. Financial problems were also common and reported more frequently among minoritized participants. Collectively, these results suggest that many survivors of COVID-19 infection continue to experience symptoms for at least 6 months after hospitalization, recovery under current care is limited, and disparities may exist in some problems after COVID-19 hospitalization.

Our findings are suggestive of prolonged sequelae that are inconsistently related to initial acuity. In line with this, a study comprising predominately outpatients with COVID-19 in Washington revealed persistent symptoms in 30% and functional impairment in 8% at a median of 6 months.^28^ A UK-based follow-up study of 1077 patients with COVID-19 hospitalized in wards and intensive care units revealed that fewer than 1 in 3 participants had recovered completely after a median 5.9 months.^1^ Together, these findings suggest a possible disconnect between the known predictors of initial COVID-19 severity and those of long-term recovery in some health domains. Alternatively, they may reflect variability in the trajectories experienced by survivors of COVID-19 owing to individual responses to treatment or differences in either inpatient or posthospital care.^29,30^

This work also fits into the broader context of research on other acute respiratory conditions. Metlay and colleagues^31^ surveyed 506 adults with acute pneumonia. They found that symptoms were still present 90 days after diagnosis. In later work among hospitalized adults, El Moussaoui and colleagues^32^ found that many respiratory symptoms resolved by 6 months, while longer-lasting deficits were associated with greater baseline age and comorbidities, which were not seen in our study. Future work should attempt to identify differences in recovery trajectories and their predictors between COVID-19 and non–COVID-19 respiratory illnesses.^33,34,35^

We found that the proportion of participants reporting cardiopulmonary symptoms increased between months 1 and 6, while the proportion of participants reporting a new ADL or IADL impairment decreased. This may reflect participants’ adaptation to symptoms over time. Alternatively, participants may have increased their activities as they exhausted work leave, caregiver help, and other support, and experienced greater symptoms as a result. Future work should also explore differences between symptom resolution and functional recovery and how these differences might relate to posthospital care or support, recurrent infection, or adaptation to functional impairments.^36,37^

Job loss, savings depletion, and forgone care occurred most often in the first month after COVID-19; yet, new bankruptcies, collection agency calls, and job changes continued to occur at 6 months and were reported more often among minoritized populations. Many COVID-19–related economic relief efforts have focused on maintaining and reopening businesses in industries hard hit by COVID-19; such efforts may fail to reach people who cannot work as a result of their illness and resulting symptoms or disabilities, which we found to be common. Extended unemployment benefits may fill this gap, but rarely last longer than 6 months, with some states providing as few as 12 weeks of benefits.^38^

Our findings add to the literature on COVID-19 recovery in several ways. First, we excluded patients with substantial pre-COVID-19 disability and compared functional limitations with preillness baselines. In doing so, our incidence estimates more closely reflect new impairments occurring after COVID-19. Second, we measured symptoms and other COVID-19–related sequelae longitudinally, enabling an understanding of how problems evolve after hospitalization in persons who survived COVID-19. Third, we enrolled a diverse cohort with varying degrees of illness across the US between August 2020 and July 2021; this reduces the risk that our findings were due to local differences in resource availability or surge conditions.

### Limitations

These findings should be considered in light of several limitations. First, participants were largely recruited from teaching hospitals and so may not reflect a broader population. This limitation is mainly important because we were unable to separate consequences of COVID-19 from consequences of the care patients received. We also relied on self-reported symptoms and comparisons with baseline health, often ascertained at follow-up. While this captures participants’ experiences of their own sequelae, it introduces the risk of misclassification as perceptions may change over time. Moreover, our incomplete response rate at each time point may have introduced bias if respondents were different from those not responding. In addition, we excluded patients with higher levels of baseline physical or cognitive dysfunction; while this was done to improve the performance of our survey instruments, it reduces generalizability to people with substantial pre-COVID-19 disability.

## Conclusions

While some individuals who survived COVID-19 experienced improvements in early posthospitalization symptoms and disability, many continued to experience cardiopulmonary, financial, and functional problems 6 months after discharge. These findings suggest the need for health care systems, clinicians, and patients to recognize and manage both persistent and new problems after COVID-19–related hospitalization.

## Notes

Supplement 1. **eTable 1.** Missingness by Variable **eTable 2.** Participant Demographic Characteristics **eTable 3.** Participant Demographics by Follow-up Status **eTable 4.** Presence of Symptoms at 6 Months by Initial Illness Severity and Comorbidity Status **eTable 5.** New or Worsened Cardiopulmonary Symptoms Over Time Compared to Pre-COVID Baseline Among Only Participants Responding to Each of Three Surveys **eTable 6.** New or Worsened Cardiopulmonary Symptoms Over Time Compared to Pre-COVID Baseline Restricted Only to Respondents With Three or More Comorbidities at Baseline (n=99) **eTable 7.** New or Worsened Cardiopulmonary Symptoms Over Time Compared to Pre-COVID Baseline Restricted to Respondents Who Received Mechanical Ventilation **eTable 8.** Predictors of New Cardiopulmonary Symptoms at 6 Months **eTable 9.** Financial Problems at Each Timepoint Among Only Participants Completing All Three Surveys **eTable 10.** Predictors of Any Financial Toxicity at 6 Months **eFigure 1.** CONSORT Diagrams—Month 1 **eFigure 2.** CONSORT Diagrams—Month 3 **eFigure 3.** CONSORT Diagrams—Month 6 **eFigure 4.** Trajectories in Perceived Recovery Over Time **eFigure 5.** Trajectories in Self-reported Fatigue **eAppendix.** List of BLUE CORAL Investigators

Supplement 2. **Nonauthor Collaborators.** The National Heart, Lung, and Blood Institute PETAL Network

Supplement 3.
Data Sharing Statement
