# Supplementary material for: Assessment of Symptom, Disability, and Financial Trajectories in Patients Hospitalized for COVID-19 at 6 Months
Source: JAMA Netw Open. 2023 Feb 14;6(2):e2255795. doi: 10.1001/jamanetworkopen.2022.55795 (PMC9929698; doi:10.1001/jamanetworkopen.2022.55795)
Supplement: Supplement 1. — eTable 1. Missingness by Variable eTable 2. Participant Demographic Characteristics eTable 3. Participant Demographics by Follow-up Status eTable 4. Presence of Symptoms at 6 Months by Initial Illness Severity and Comorbidity Status eTable 5. New or Worsened Cardiopulmonary Symptoms Over Time Compared to Pre-COVID Baseline Among Only Participants Responding to Each of Three Surveys eTable 6. New or Worsened Cardiopulmonary Symptoms Over Time Compared to Pre-COVID Baseline Restricted Only to Respondents With Three or More Comorbidities at Baseline (n=99) eTable 7. New or Worsened Cardiopulmonary Symptoms Over Time Compared to Pre-COVID Baseline Restricted to Respondents Who Received Mechanical Ventilation eTable 8. Predictors of New Cardiopulmonary Symptoms at 6 Months eTable 9. Financial Problems at Each Timepoint Among Only Participants Completing All Three Surveys eTable 10. Predictors of Any Financial Toxicity at 6 Months eFigure 1. CONSORT Diagrams—Month 1 eFigure 2. CONSORT Diagrams—Month 3 eFigure 3. CONSORT Diagrams—Month 6 eFigure 4. Trajectories in Perceived Recovery Over Time eFigure 5. Trajectories in Self-reported Fatigue eAppendix. List of BLUE CORAL Investigators [file jamanetwopen-e2255795-s001.pdf]

Supplementary Online Content

Admon AJ, Iwashyna TJ, Kamphuis LA, et al; National Heart, Lung, and Blood Institute PETAL Network. Assessment of symptom, disability, and financial trajectories in patients hospitalized for COVID-19 at 6 months. *JAMA Netw Open*. 2023;6(2):e2255795. doi:10.1001/jamanetworkopen.2022.55795

eTable 1. Missingness by Variable

eTable 2. Participant Demographic Characteristics

eTable 3. Participant Demographics by Follow-up Status

eTable 4. Presence of Symptoms at 6 Months by Initial Illness Severity and Comorbidity Status

eTable 5. New or Worsened Cardiopulmonary Symptoms Over Time Compared to Pre-COVID Baseline Among Only Participants Responding to Each of Three Surveys

eTable 6. New or Worsened Cardiopulmonary Symptoms Over Time Compared to Pre-COVID Baseline Restricted Only to Respondents With Three or More Comorbidities at Baseline (n=99)

eTable 7. New or Worsened Cardiopulmonary Symptoms Over Time Compared to Pre-COVID Baseline Restricted to Respondents Who Received Mechanical Ventilation

eTable 8. Predictors of New Cardiopulmonary Symptoms at 6 Months

eTable 9. Financial Problems at Each Timepoint Among Only Participants Completing All Three Surveys

eTable 10. Predictors of Any Financial Toxicity at 6 Months

eFigure 1. CONSORT Diagrams—Month 1

eFigure 2. CONSORT Diagrams—Month 3

eFigure 3. CONSORT Diagrams—Month 6

eFigure 4. Trajectories in Perceived Recovery Over Time

eFigure 5. Trajectories in Self-reported Fatigue

eAppendix. List of BLUE CORAL Investigators

This supplementary material has been provided by the authors to give readers additional information about their work.

© 2023 Admon AJ et al. *JAMA Network Open.*

eTable 1: Missingness by variable

Variable	Number Missing	Percent Missing
**Financial Problems**		
Used up all or most of savings	36	1.8%
Were unable to pay for necessities	30	1.5%
Were contacted by a collection agency	36	1.8%
Declared bankruptcy	34	1.7%
Skipped or delayed medical care because of cost	30	1.5%
Took less medications because of the cost	32	1.6%
Lost a job	21	1.1%
Had to change work	29	1.5%
Had a loved one take time off of work	27	1.4%
Been told that insurance would not cover therapy or rehab	40	2.0%
Been told that insurance would not cover equipment	41	2.1%
How much of a drain on your family's financial resources	36	1.8%
**Symptoms**		
Cough	33	1.7%
Chest trouble with smells/fumes	41	2.1%
Breathlessness with sleep	38	1.9%
Chest trouble when upset	37	1.9%
Chest trouble getting around the house	36	1.8%
Chest trouble leads to going home early	42	2.1%
Breathlessness when laughing	40	2.0%
Using home oxygen	33	1.7%
Using CPAP or another breathing machine during sleep	37	1.9%
Swelling in feet, ankles, or legs	52	2.7%
Breathlessness when lying flat	43	2.2%
Rapid or irregular heartbeat	57	2.9%
Chest pain, tightness, or angina	47	2.4%
**ADL/IADL deficits versus enrollment**	7	0.4%
**Feeling tired or having little energy**	140	7.1%
**Quality of Life (EQ-5D-5L)**		
Anxiety/Depression	12	0.6%
Mobility	5	0.3%
Pain/Discomfort	8	0.4%
Self-care	8	0.4%
Usual activities	11	0.6%
**Demographics**		
Race/Ethnicity	0	0.0%
Medicare Beneficiary	8	0.4%
Primary Language	3	0.2%
**Severity of Illness**		
Charlson comorbidities	0	0.0%
Discharge disposition		
Immunosuppressed status	0	0.0%
Hospital length of stay	0	0.0%
Highest level of care	0	0.0%
Maximal oxygen use	0	0.0%
Vasopressors	0	0.0%

CPAP: continuous positive airway pressure; EQ-5D-5L: European Quality of Life five-dimension five-level instrument

© 2023 Admon AJ et al. *JAMA Network Open.*

eTable 2: Participant demographic characteristics (n=825)

Characteristic	No. (%)
**Age, median (IQR), y**	56 (43, 66)
**Age, categorical, y**	
≤ 50	313 (38.9)
51-60	169 (21.0)
61-70	191 (23.7)
71-80	87 (10.8)
≥ 81	45 (5.6)
**Sex**	
Male	444 (54.0)
Female	379 (46.1)
**Race**	
Hispanic	253 (30.7)
Non-Hispanic Black or African American	145 (17.6)
Non-Hispanic White	360 (43.6)
Other or Unknown*	67 (8.1)
**Primary Language**	
English	717 (86.9)
Spanish	108 (13.1)
**Charlson Comorbidities**	
None	444 (53.8)
1	186 (22.6)
2	96 (11.6)
3 or more	99 (12.0)
**Immunocompromised**	123 (14.9)
**Highest Level of Care**	
Ward	540 (65.5)
Intermediate or stepdown	185 (22.4)
Intensive care unit	100 (12.1)
**Maximal oxygen support**	
None	134 (16.2)
Conventional oxygen	441 (53.5)
High flow nasal cannula	131 (15.9)
Non-invasive ventilation	43 (5.2)
Invasive mechanical ventilation	76 (9.2)
**Vasopressor Use**	71 (8.6)
**Length of stay, median (IQR), d**	6 (4, 10)
**Hospital discharge location****	
Home independently	640 (77.6)
Home with help	120 (14.6)
To another facility	65 (7.9)

*Includes American Indian/Alaskan Native, Asian, Native Hawaiian/Pacific Islander, other/declined, and unknown.
**Home discharge without services includes discharge against medical advice and discharge to a COVID Hotel or similar non-healthcare facility.

© 2023 Admon AJ et al. *JAMA Network Open.*

eTable 3: Participant demographic by follow-up status

Characteristic	Completed only Month 1 (n=84)	Completed Months 1, 3, and 6 (n=473)	*P* Value[†]
**Age, median (IQR), y**	51 (38, 65)	59 (47, 68)	<0.001
**Age, categorical, y**			0.005
≤ 50	38 (46.9%)	143 (31.0%)	
51-60	17 (21.0%)	105 (22.7%)	
61-70	22 (27.2%)	123 (26.6%)	
≥71	4 (4.9%)	91 (18.7%)	
**Sex**			0.87
Male	40 (47.6%)	230 (48.6%)	
Female	44 (52.4%)	243 (51.4%)	
**Race**			0.14
Hispanic	31 (38.1%)	127 (26.9%)	
Non-Hispanic Black or African American	11 (13.1%)	87 (18.4%)	
- Non-Hispanic White	34 (40.5%)	227 (48.0%)	
Other or Unknown*	7 (8.3%)	32 (6.8%)	
**Primary Language**			0.61
English	72 (85.7%)	415 (87.7%)	
Spanish	12 (14.3%)	58 (13.3%)	
**Charlson Comorbidities**			0.12
None	52 (61.9%)	241 (51.0%)	
1	12 (14.3%)	121 (25.6%)	
2	11 (13.1%)	53 (11.2%)	
3 or more	9 (10.7%)	58 (12.3%)	
**Immunocompromised**	12 (14.3%)	72 (15.2%)	0.83
**Highest Level of Care**			0.52
Ward	60 (71.4%)	309 (65.3%)	
Intermediate or stepdown	16 (19.1%)	103 (21.8%)	
Intensive care unit	8 (9.5%)	61 (12.9%)	
**Maximal oxygen support**			0.26
None	20 (23.8%)	68 (14.4%)	
Conventional oxygen	42 (50.0%)	257 (54.3%)	
High flow nasal cannula	12 (14.3%)	79 (16.7%)	
Non-invasive ventilation	3 (3.6%)	28 (5.9%)	
Invasive mechanical ventilation	7 (8.3%)	41 (8.7%)	
**Vasopressor Use**	10 (11.9%)	35 (7.4%)	0.16
**Length of stay, median (IQR), d**	5 (3, 8)	5 (3, 9)	0.09
**Hospital discharge location****			0.48
Home independently	68 (81.0%)	354 (74.8%)	
Home with help	11 (13.1%)	79 (16.7%)	
To another facility	5 (6.0%)	40 (8.5%)	

*Includes American Indian/Alaskan Native, Asian, Native Hawaiian/Pacific Islander, other/declined, and unknown.
**Home discharge without services includes discharge against medical advice and discharge to a COVID Hotel or similar non-healthcare facility.
[†]Using Wilcoxon rank-sum test for continuous variables and chi-squared test for categorical variables.

© 2023 Admon AJ et al. *JAMA Network Open.*

eTable 4: Presence of symptoms at 6 months by initial illness severity and comorbidity status.

	All participants at 6 months (n=641)	Ward only (n=418)	ICU (n=82)	Zero comorbidities (n=329)	At least one comorbidity (n=311)	3 or More comorbidities (n=81)	Diabetes Mellitus (n=198)	Chronic Lung Disease (n=45)
Any cardiorespiratory symptom at 6 months	75.4% (71.9%, 78.8%)	76.0% (71.6%, 80.0%)	76.8% (66.2%, 85.4%)	72.0% (66.8%, 76.7%)	79.1% (74.2%, 83.5%)	81.5% (71.3%, 89.2%)	79.8% (73.5%, 85.2%)	88.9% (76.0%, 96.3%)
Any financial problem at 6 months	56.4% (52.5%, 60.3%	57.6% (52.7%, 62.3%)	47.6% (36.4%, 58.9%)	51.7% (46.1%, 57.2%)	61.4% (55.8%, 66.9%)	54.3% (42.9%, 65.4%)	61.9% (54.8%, 68.7%)	62.2% (46.5%, 76.2%)
At least 1 new ADL/IADL impairment at 6 months	47.3% (43.3%, 51.2%)	44.3% (39.4%, 49.2%)	40.2% (30.0%, 51.7%)	40.9% (35.6%, 46.4%)	54.0% (48.3%, 60.0%)	51.9% (40.5%, 63.1%)	52.3% (45.1%, 59.4%)	62.2% (46.5%, 76.2%)
Less than 100% of baseline health at 6 months	71.8% (68.2%, 75.3%)	70.8% (66.1%, 75.2%)	66.7% (55.3%, 76.8%)	69.8% (64.4%, 74.7%)	74.0% (68.7%, 78.8%)	74.4% (63.6%, 83.4%)	71.8% (64.9%, 78.0%)	68.9% (53.4%, 81.8%)

ADL: Activity of daily living; IADL: Instrumental activity of daily living

© 2023 Admon AJ et al. *JAMA Network Open.*

eTable 5: New or worsened cardiopulmonary symptoms over time compared to pre-COVID baseline among only participants responding to each of three surveys (n=473 participants)

	Survey Timepoint (%)			
	Month 1	Month 3	Month 6	*P* Value*
**Symptoms**				
Chest trouble with odors/fumes	13.58	30.54	29.85	<0.001
Cough	20.82	21.79	27.72	0.006
Swelling in feet, ankles, or legs	10.99	22.48	24.78	<0.001
Chest trouble when upset	16.81	26.55	24.52	<0.001
Rapid or irregular heartbeat	19.52	21.43	20.00	0.82
Breathlessness with sleep	14.19	19.91	21.87	<0.001
Chest pain, tightness, or angina	14.87	19.23	18.75	0.06
Breathlessness when laughing	18.24	17.09	17.95	0.89
Breathlessness when lying flat	12.90	13.68	13.95	0.57
Chest trouble getting around the house	15.91	15.42	16.14	0.91
Chest trouble leads to going home early	15.09	16.03	15.07	0.99
**Any symptom**	53.78	64.73	64.68	<0.001
**CPAP and supplemental oxygen**				
Using CPAP or another breathing machine during sleep	5.64	13.43	15.07	<0.001
Using home oxygen	21.20	15.35	12.53	<0.001
**Any CPAP or supplemental oxygen**	23.44	23.67	23.14	0.88
**Any symptom, CPAP, or supplemental oxygen**	68.74	75.74	76.48	0.002

**P* value calculating using Somers' D accounting for clustering of observations by respondent.
CPAP: continuous positive airway pressure

© 2023 Admon AJ et al. *JAMA Network Open.*

eTable 6: New or worsened cardiopulmonary symptoms over time compared to pre-COVID baseline restricted only to respondents with three or more comorbidities at baseline (N=99)

	Survey Timepoint (%)			
	Month 1	Month 3	Month 6	*P* Value*
**Symptoms**				
Chest trouble with odors/fumes	11.25	17.57	24.05	0.01
Cough	11.25	24.00	23.75	0.005
Swelling in feet, ankles, or legs	8.86	23.61	23.08	0.001
Chest trouble when upset	12.50	18.92	17.72	0.29
Rapid or irregular heartbeat	12.66	13.70	12.99	0.94
Breathlessness with sleep	11.25	14.67	20.99	0.08
Chest pain, tightness, or angina	8.86	13.33	11.69	0.51
Breathlessness when laughing	17.72	8.22	12.82	0.33
Breathlessness when lying flat	10.13	10.81	16.67	0.10
Chest trouble getting around the house	15.19	17.57	16.05	0.84
Chest trouble leads to going home early	8.97	6.76	7.50	0.73
**Any symptom**	43.04	63.89	61.25	0.12
**CPAP and supplemental oxygen**				
Using CPAP or another breathing machine during sleep	3.85	13.33	17.50	<0.001
Using home oxygen	26.58	20.00	18.75	0.17
**Any CPAP or supplemental oxygen**	29.11	28.00	30.00	0.88
**Any symptom, CPAP, or supplemental oxygen**	71.25	80.00	81.48	0.006

**P* value calculating using Somers' D accounting for clustering of observations by respondent.
CPAP: continuous positive airway pressure

© 2023 Admon AJ et al. *JAMA Network Open.*

eTable 7: New or worsened cardiopulmonary symptoms over time compared to pre-COVID baseline restricted only to respondents who received mechanical ventilation (N=76)

	Survey Timepoint (%)			
	Month 1	Month 3	Month 6	*P* Value*
**Symptoms**				
Chest trouble with odors/fumes	8.93	18.97	27.78	0.005
Cough	19.30	31.58	29.63	0.12
Swelling in feet, ankles, or legs	14.29	28.07	16.98	0.63
Chest trouble when upset	17.86	24.56	23.64	0.38
Rapid or irregular heartbeat	16.07	19.64	26.42	0.12
Breathlessness with sleep	12.28	17.54	23.21	0.10
Chest pain, tightness, or angina	8.93	17.54	19.23	0.006
Breathlessness when laughing	16.07	26.32	25.93	0.15
Breathlessness when lying flat	16.07	17.86	3.70	0.001
Chest trouble getting around the house	10.71	12.73	18.18	0.15
Chest trouble leads to going home early	16.07	14.55	17.86	0.77
**Any symptom**	50.00	73.68	62.96	0.06
**CPAP and supplemental oxygen**				
Using CPAP or another breathing machine during sleep	12.28	10.34	18.18	0.28
Using home oxygen	36.84	19.30	23.64	0.05
**Any CPAP or supplemental oxygen**	43.86	24.56	34.55	0.19
**Any symptom, CPAP, or supplemental oxygen**	79.31	81.36	73.21	0.41

**P* value calculating using Somers' D accounting for clustering of observations by respondent.
CPAP: continuous positive airway pressure

© 2023 Admon AJ et al. *JAMA Network Open.*

eTable 8: Predictors of new cardiopulmonary symptoms at 6 months (N=523 participants). Model 1 includes demographic characteristics. Model 2 adds pre-hospital comorbidities. Model 3 adds hospital severity of illness. Model 4 adds presence of cardiopulmonary symptoms at 1 month.

	Odds Ratio (95% CI)			
	Model 1	Model 2	Model 3	Model 4
Age in Decades	2.28*	2.18*	2.03	1.74
	(1.14, 4.55)	(1.05 4.52)	(0.92, 4.45)	(0.76, 4.02)
Age^2	0.94*	0.94*	0.94	0.95
	(0.88, 0.99)	(0.88 1.00)	(0.87, 1.01)	(0.88, 1.03)
Sex (vs. female)				
Male	0.48**	0.48**	0.50**	0.59*
	(0.31, 0.76)	(0.31 0.77)	(0.32, 0.79)	(0.37, 0.95)
Race and Ethnicity (vs. non-Hispanic White)				
Hispanic	1.01	0.98	0.94	1.11
	(0.66, 1.54)	(0.65 1.47)	(0.61, 1.46)	(0.67, 1.82)
Non-Hispanic Black	1.22	1.19	1.22	1.62
	(0.70, 2.12)	(0.70 2.02)	(0.71, 2.08)	(0.94, 2.80)
Other/Unknown†	0.61	0.56*	0.55*	0.83
	(0.36, 1.02)	(0.32 1.00)	(0.31, 0.98)	(0.39, 1.76)
Medicare beneficiary (vs. non-Medicare beneficiary)				
Yes	0.88	1.49	0.88	0.94
	(0.54, 1.45)	(0.79 2.81)	(0.52, 1.47)	(0.51, 1.72)
Unknown	1.49	0.86	1.62	1.67
	(0.79, 2.81)	(0.51 1.43)	(0.87, 3.03)	(0.87, 3.21)
Number of Charlson comorbidities (vs. 0)				
1		1.14	1.12	1.24
		(0.66 1.97)	(0.63, 2.01)	(0.57, 2.72)
2		2.39*	2.37*	2.13
		(1.06 5.40)	(1.08, 5.20)	(0.83, 5.45)
3 or more		1.74	1.68	1.50
		(0.83 3.64)	(0.79, 3.59)	(0.76, 2.96)
Immunocompromised (vs. not immunocompromised)				
Immunocompromised		0.79	0.91	1.10
		(0.48 1.30)	(0.55, 1.51)	(0.66, 1.84)
Quartile of Length of Stay (vs. first quartile)				
Second quartile			0.87	0.82
			(0.50, 1.53)	(0.47, 1.43)
Third quartile			1.59	1.29
			(0.89, 2.85)	(0.76, 2.19)
Fourth (longest) quartile			1.37	1.18
			(0.68, 2.75)	(0.58, 2.38)
Highest Level of Care (vs. ward)				
Intermediate or Stepdown			0.51	0.52
			(0.22, 1.16)	(0.20, 1.33)
ICU			0.84	0.84
			(0.42, 1.68)	(0.34, 2.07)
Maximal oxygen device (vs. none)				
Conventional Oxygen			1.83*	1.71*
			(1.13, 2.97)	(1.10, 2.66)
High Flow Nasal Cannula			1.95	1.74
			(0.89, 4.26)	(0.76, 3.95)
Non-invasive Positive Pressure Ventilation			3.54	2.49
			(0.99, 12.63)	(0.63, 9.85)
Mechanical Ventilation			1.54	1.73
			(0.28, 8.63)	(0.22, 13.76)
Vasopressors (vs no vasopressors)				
Received Vasopressors			1.72	1.01
			(0.77, 3.87)	(0.30, 3.40)
Cardiorespiratory symptoms at 1 month (vs. no symptoms)				
Yes				3.25***
				(2.01, 5.27)
Intercept	0.45	0.50	0.36	0.22
	(0.06, 3.24)	(0.06 3.92)	(0.04, 2.97)	(0.02, 2.24)
AUROC	0.625	0.643	0.677	0.721

*** p<.001, ** p<.01, * p<.05,

†Includes American Indian/Alaskan Native, Asian, Native Hawaiian/Pacific Islander, other/declined, and unknown

ICU: intensive care unit; AUROC: area under the receiver operating characteristic curve

© 2023 Admon AJ et al. *JAMA Network Open.*

eTable 9: Financial problems at each timepoint among only participants responding to each of three surveys (n=473 participants)

	Survey Timepoint (%)			
	Month 1	Month 3	Month 6	*P* Value*
**At any time because of COVID hospitalization, have you…**				
used up all or most of savings?	29.21	31.26	33.40	0.05
been unable to pay for necessities?	17.48	18.55	19.40	0.33
been contacted by a collection agency?	10.23	11.99	16.70	<0.001
skipped or delayed medical care because of cost?	7.46	8.49	8.32	0.51
taken less medications because of the cost?	4.90	6.61	6.40	0.25
declared bankruptcy?	0.85	1.28	1.50	0.35
**Since our last contact, have you…**				
had a loved one take time off of work?	38.54	23.62	20.51	<0.001
had to change work?	13.25	14.07	10.59	0.13
been told that insurance would not cover therapy or rehab?	3.85	5.59	6.17	0.06
lost a job?	7.46	6.57	6.34	0.42
been told that insurance would not cover equipment?	6.42	5.59	4.71	0.17
**Prevalence of any financial problem**	63.83	56.36	53.70	<0.001

**P* value calculating using Somers' D accounting for clustering of observations by respondent.

© 2023 Admon AJ et al. *JAMA Network Open.*

eTable 10: Predictors of any financial problems at 6 months (N=528 participants). Model 1 includes demographic characteristics. Model 2 adds pre-hospital comorbidities. Model 3 adds hospital severity of illness. Model 4 adds presence of financial problems at 1 month.

	Odds Ratio (95% CI)			
	Model 1	Model 2	Model 3	Model 4
Age in Decades	1.80<br>(0.90, 3.58)	1.56<br>(0.76 3.20)	1.51<br>(0.73, 3.12)	1.46<br>(0.68, 3.13)
Age^2	0.94<br>(0.88, 1.00)	0.95<br>(0.89 1.01)	0.95<br>(0.89, 1.02)	0.96<br>(0.90, 1.03)
Sex (vs. female)				
Male	0.80<br>(0.61, 1.06)	0.82<br>(0.61 1.10)	0.82<br>(0.61, 1.11)	0.77<br>(0.52, 1.14)
Race and Ethnicity (vs. non-Hispanic White)				
Hispanic	3.64***<br>(2.51, 5.27)	3.63***<br>(2.50 5.27)	3.52***<br>(2.41, 5.15)	3.74***<br>(2.24, 6.23)
Non-Hispanic Black	2.99***<br>(2.01, 4.44)	2.86***<br>(1.94 4.20)	2.97***<br>(2.10, 4.20)	2.63***<br>(1.65, 4.20)
Other/Unknown[†]	2.66**<br>(1.28, 5.55)	2.53*<br>(1.19 5.41)	2.47*<br>(1.19, 5.12)	3.28*<br>(1.32, 8.17)
Medicare beneficiary (vs. non-Medicare Beneficiary)				
Yes	0.75<br>(0.52, 1.09)	1.46<br>(0.90 2.36)	0.70<br>(0.48, 1.01)	0.89<br>(0.53, 1.51)
Unknown	1.54<br>(0.96, 2.49)	0.69*<br>(0.47 1.00)	1.59<br>(0.90, 2.83)	1.35<br>(0.68, 2.69)
Number of Charlson comorbidities (vs. 0)				
1		1.83**<br>(1.27 2.64)	1.84**<br>(1.24, 2.74)	1.64<br>(0.99, 2.73)
2		2.08**<br>(1.34 3.24)	2.08**<br>(1.27, 3.38)	1.68<br>(0.92, 3.08)
3 or more		1.25<br>(0.83 1.89)	1.25<br>(0.75, 2.08)	1.27<br>(0.67, 2.42)
Immunocompromised (vs. not immunocompromised)				
Immunocompromised		1.21<br>(0.79 1.86)	1.19<br>(0.78, 1.84)	1.13<br>(0.67, 1.89)
Quartile of Length of Stay (vs. first quartile)				
Second quartile			1.04<br>(0.57, 1.87)	1.01<br>(0.51, 2.00)
Third quartile			1.57<br>(0.84, 2.93)	1.22<br>(0.64, 2.35)
Fourth (longest) quartile			1.80<br>(0.88, 3.69)	1.44<br>(0.61, 3.43)
Highest Level of Care (vs. ward)				
Intermediate or Stepdown			0.59<br>(0.31, 1.12)	0.58<br>(0.30, 1.14)
ICU			0.80<br>(0.42, 1.54)	0.66<br>(0.35, 1.22)
Maximal oxygen device (vs. none)				
Conventional Oxygen			0.82<br>(0.47, 1.43)	0.70<br>(0.35, 1.41)
High Flow Nasal Cannula			0.96<br>(0.42, 2.19)	0.65<br>(0.24, 1.77)
Non-invasive Positive Pressure Ventilation			0.80<br>(0.35, 1.85)	0.72<br>(0.35, 1.50)
Mechanical Ventilation			0.55<br>(0.11, 2.73)	0.90<br>(0.11, 7.61)
Vasopressors (vs. no vasopressors)				
Received Vasopressors			2.65<br>(0.69, 10.13)	1.11<br>(0.20, 6.05)
Financial problems at 1 month (vs. no financial problems)				
Yes				8.03***<br>(5.12, 12.61)
Intercept	0.23<br>(0.04, 1.39)	0.30<br>(0.05 1.89)	0.36<br>(0.06, 2.36)	0.11*<br>(0.01, 0.92)
AUROC	0.692	0.707	0.722	0.811

*** p<.001, ** p<.01, * p<.05

[†]Includes American Indian/Alaskan Native, Asian, Native Hawaiian/Pacific Islander, other/declined, and unknown.

ICU: intensive care unit; AUROC: area under the receiver operating characteristic curve

© 2023 Admon AJ et al. *JAMA Network Open.*

eFigure 1: CONSORT Diagram – Month 1

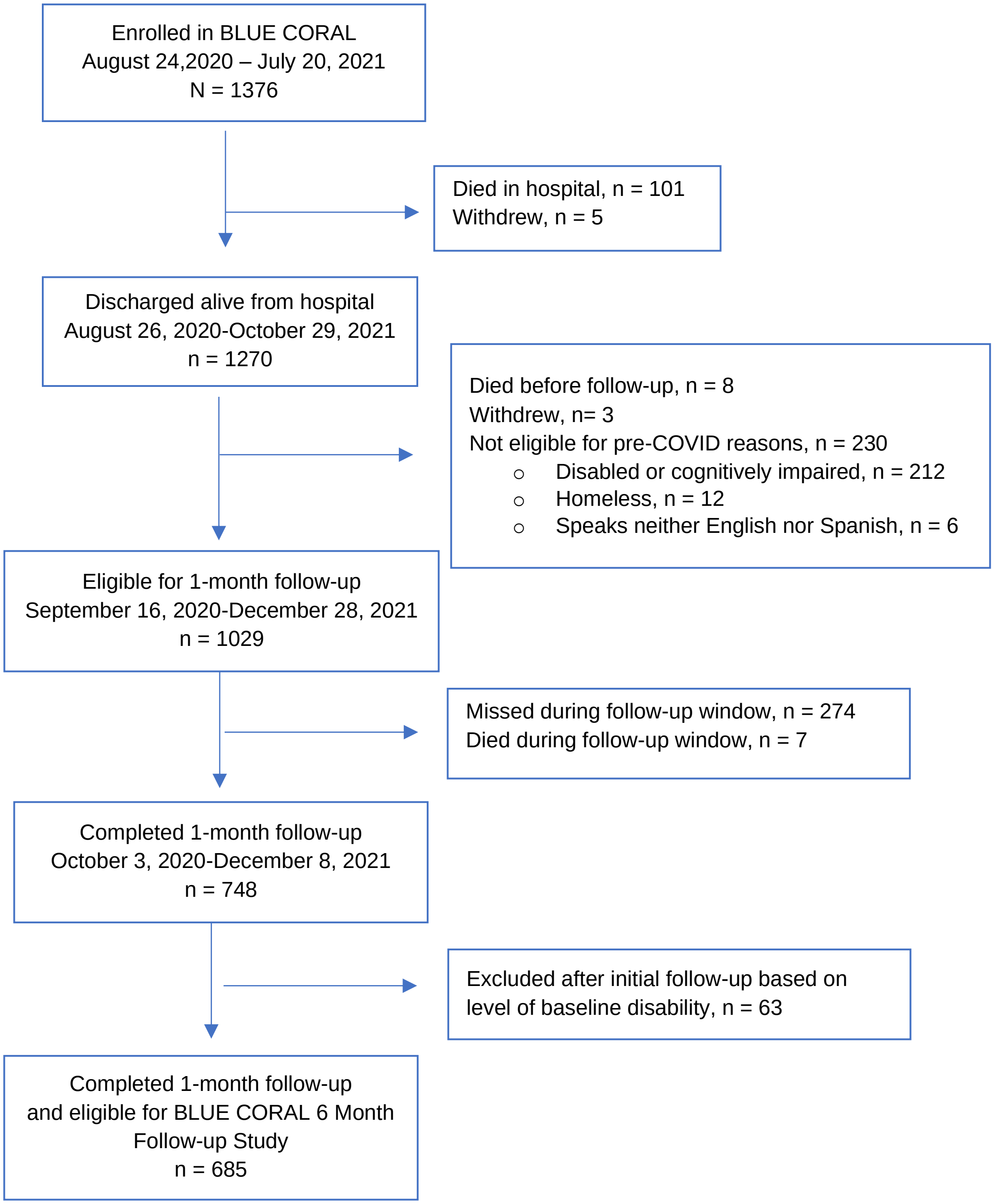

© 2023 Admon AJ et al. *JAMA Network Open.*

eFigure 2: CONSORT Diagram – Month 3

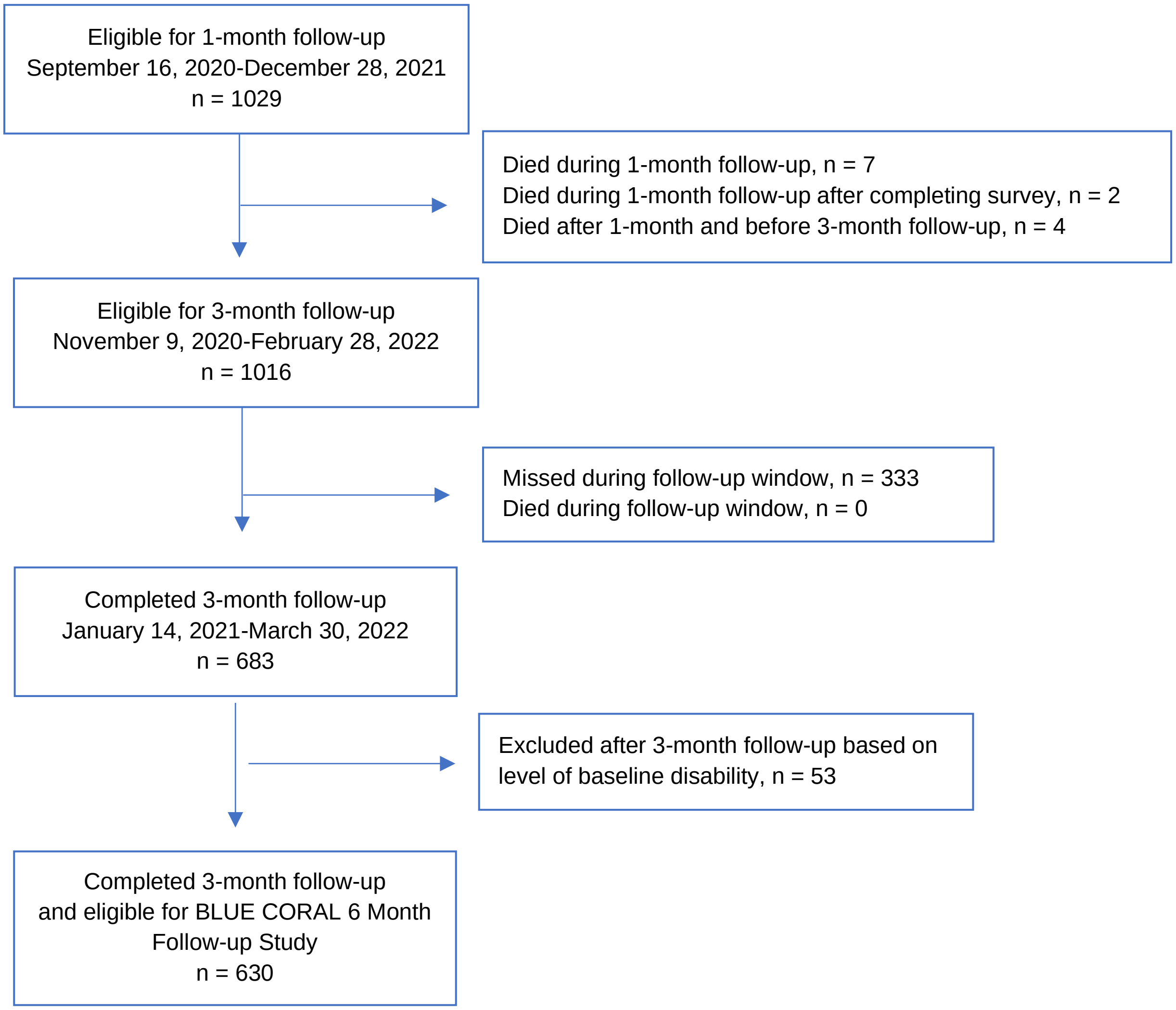

© 2023 Admon AJ et al. *JAMA Network Open.*

eFigure 3: CONSORT Diagrams – Month 6

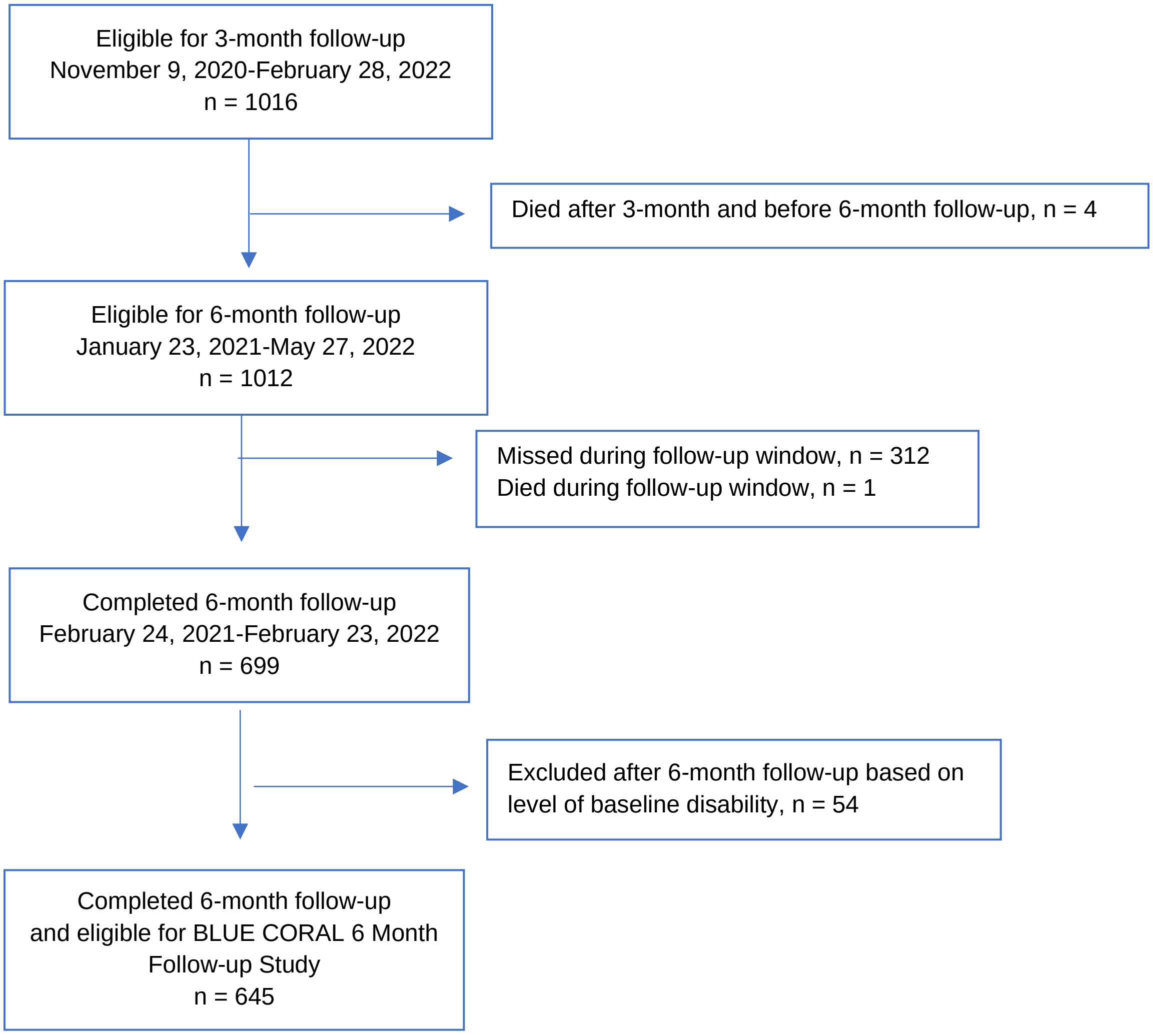

© 2023 Admon AJ et al. *JAMA Network Open.*

eFigure 4: Trajectories in perceived recovery over time. Alluvial diagram displaying perceived recovery (as a percentage of baseline health) of respondents at each timepoint (vertical bars) and changes in responses over time (horizontal lines). Bar and line thicknesses are proportional to the number of patients represented by the relevant category or change.

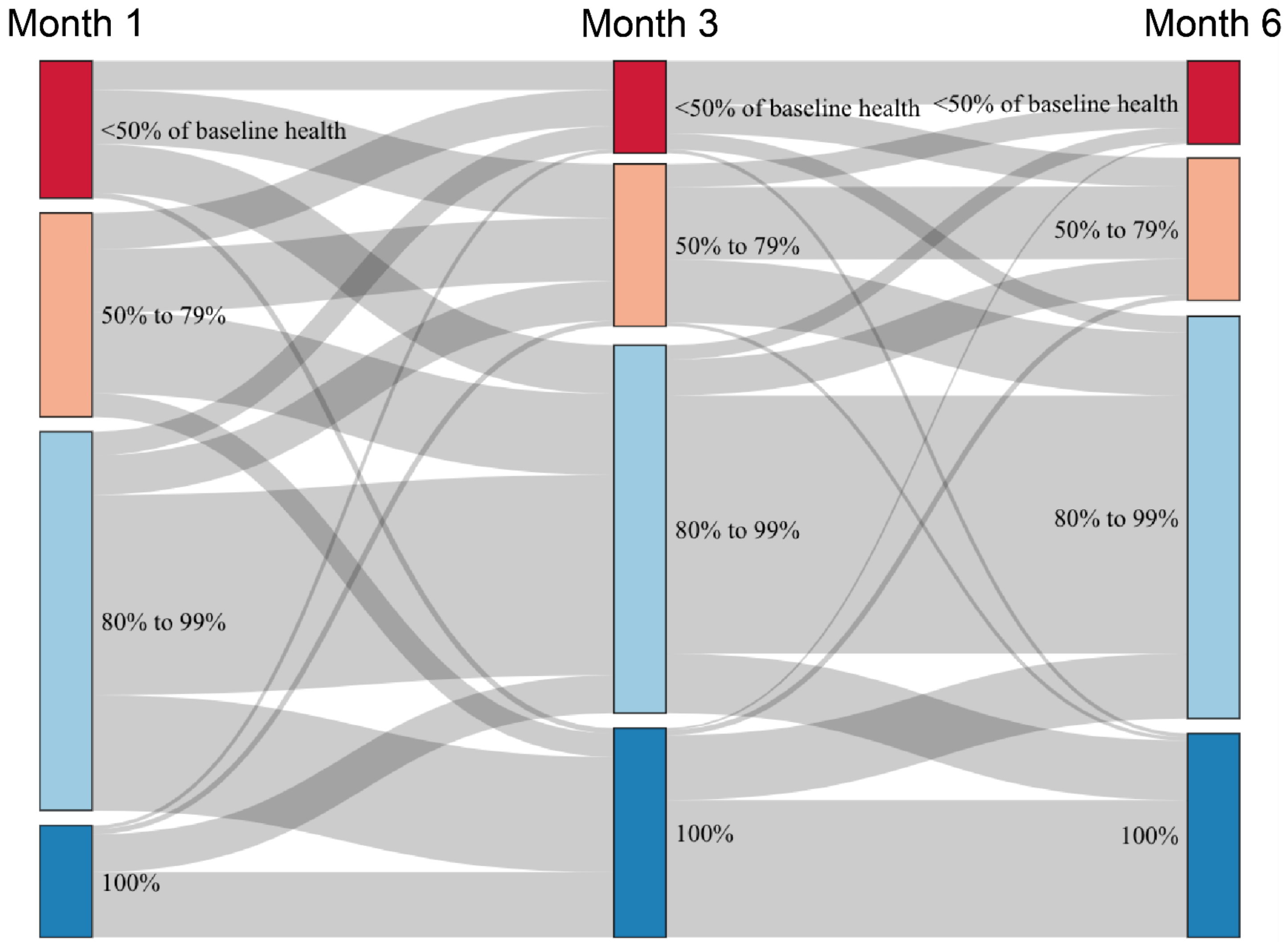

© 2023 Admon AJ et al. *JAMA Network Open.*

eFigure 5: Trajectories in self-reported fatigue. Alluvial diagram displaying how often a participant reported fatigue over the preceding 14 days (vertical bars) and changes in responses over time (horizontal lines). Bar and line thicknesses are proportional to the number of patients represented by the relevant category or change.

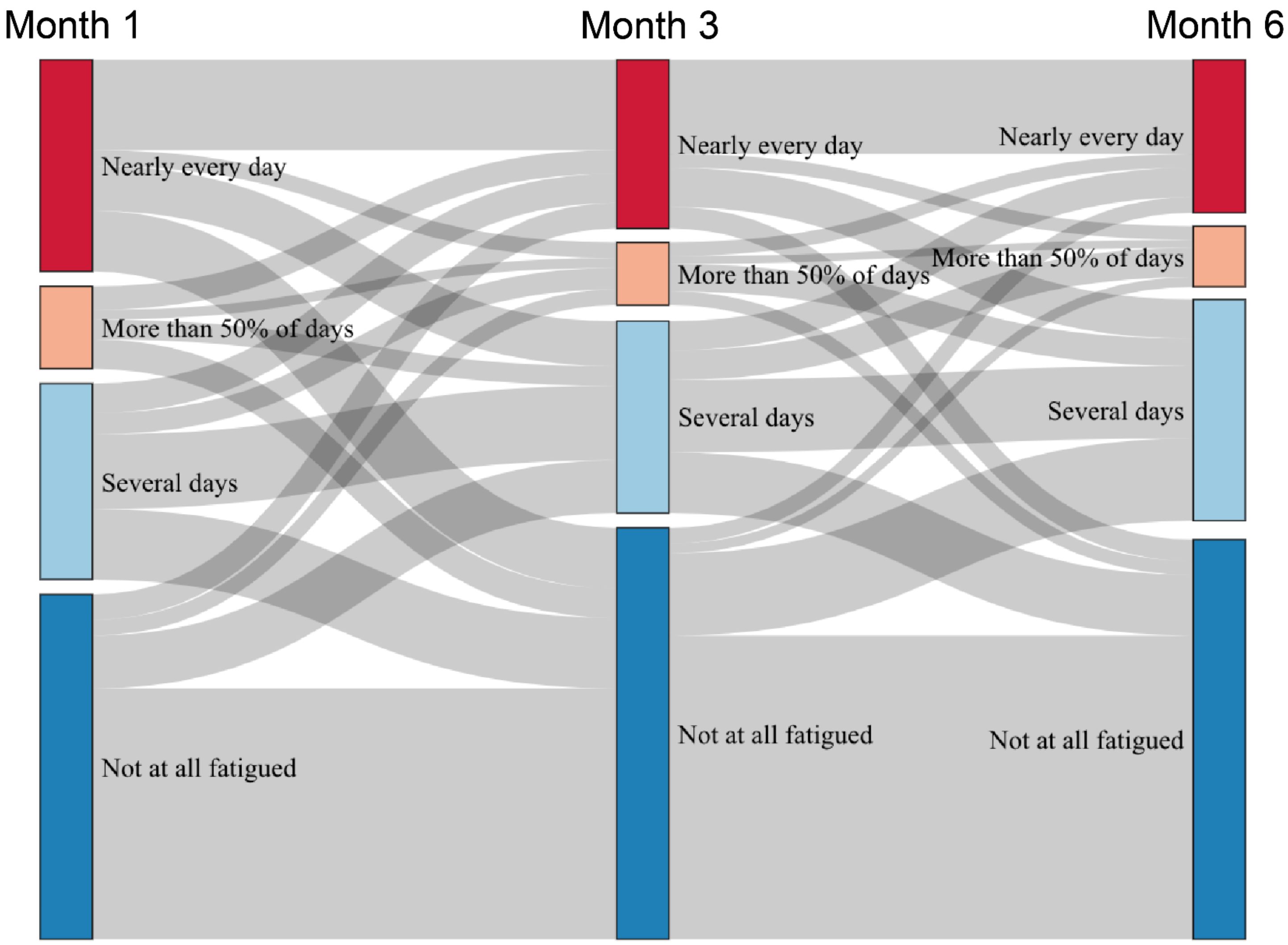

© 2023 Admon AJ et al. *JAMA Network Open.*

Complete list of BLUE CORAL Collaborators

Biology and Longitudinal Epidemiology of PETAL COVID-19 Observational Study (BLUE CORAL):

ALIGNE Clinical Center: Baystate Medical Center -Jay Steingrub*, Mark Tidswell, Lori Kozikowski, Lesley De Souza, Cynthia Kardos, Sarah Romain; Brigham and Women's Hospital -Rebecca M. Baron, Mayra Pinilla, Katherin Zambrano-Vera, Antonio Arciniegas; Maine Medical Center -Richard Riker, Christine Lord, Ashley Eldridge; University of Florida: Marie-Carmelle Elie, Torben K Becker, Matthew Shaw, Christopher J Duncan, Jesseca Antoine

BOSTON Clinical Center: Beth Israel Deaconess Medical Center -Daniel Talmor*, Nathan Shapiro*, Valerie Banner-Goodspeed, Sharon Hayes, Krystal Capers, Andre de Souza Licht, Lisa Kurt; Massachusetts General Hospital -Kathryn Hibbert, Kelsey Brait, Tatiana Held; University of Mississippi Medical Center -Alan E Jones**, Rebekah Peacock, James W. Galbraith, Utsav Nandi, Jenna L. Davis**

CALIFORNIA Clinical Center: University of California San Francisco -Michael Matthay*, Kirsten Kangelaris, Kimberly Yee, Kimia Ashktorab, Rachel Gropper, Anika Agrawal; University of California San Francisco – Fresno -Eyad Almasri, Kinsley Hubel, Mohamed Fayed, Alyssa Hughes, Rebekah Garcia; Stanford University Hospital -Angela Rogers, Jennifer Wilson, Rosemary Vojnik, Jonasel Roque, Cynthia Perez; UCLA Ronald Reagan Medical Center -Nida Qadir, George Lim, Steve Chang, Greg Hendey*, Rebecca Beutler, Trisha Agarwal, Julia Vargas; University of Texas Health Science Center at Houston -Ryan Huebinger, Bela Patel, Elizabeth Vidales

COLORADO Clinical Center: University of Colorado Hospital -Marc Moss*, Adit Ginde*, Neil Aggarwal*, Jeffrey McKeehan, Carrie Higgins, Lani Finck; Denver Health Medical Center -Jason Haukoos, Ivor Douglas, Carolynn Lyle, Stephanie Gravitz, Terra Hiller, Ann Z Garcia, Alicia K Cupelo, Judy L Oakes; National Jewish Health|St. Joseph Hospital -James Finigan, Christine Griesmer, Cole Waliczek

MICHIGAN Clinical Center: University of Michigan Medical Center -Robert C. Hyzy*, Pauline K. Park*, Kristine Nelson, Norman Olbrich, Stephen Kay; Henry Ford Health System -Namita Jayaprakash, Emanuel P Rivers, Jennifer Swiderek, Jasreen Kaur Gill, Sheri Renaud, Ariella Hodari Gupta, Jacqueline Day, Anja Kathrina Jaehne, Aaron Cook, Jayna Gardner-Gray; Sinai Grace Hospital -Robert Sherwin, James Wooden, Thomas Mazzocco, Jacob Winkel; Detroit Receiving Hospital & Harper University Hospital -Robert Sherwin, Thomas Mazzocco, Theodore Falcon, Jeffrey Harrison; FUNCTION Group - Lee Kamphuis, Steve Vaughn, Sarah Seelye, Stacy Anderson, Taylor Bernstein, Aissa Cabrales, Jennifer Cano, Emily DelMonico, Dana Horowitz, Peggy Korpela, Max Monahan, Tiffany Lee, Jessie Milne, Stephanie Ortiz Chavez, Marylena Rouse, Melanie Vandersluis

MONTEFIORE-SINAI Clinical Center: Montefiore Medical Center -Michelle Gong, Aluko Hope, Omowunmi Amosu, Hiwet Tzehaie, Sabah Boujid, Martha Torres; Mt. Sinai -Lynne D. Richardson, Kusum Mathews, Sam Acquah, Patrick Maher, Neha Goel, George Loo; Montefiore Medical Center - Weiler -Tina Chen, Rahul Nair, Brenda Lopez, Manuel Hache Marliere, Marjan Islam; University of Arizona -Jarrod Mosier, Cameron Hypes, Ariana Cano, Boris Gilson, Elizabeth Salvaggio Campbell

OHIO Clinical Center: Cleveland Clinic Foundation -Abhijit Duggal*, Omar Mehkri, Kiran Ashok, Siddharth P. Dugar, Simon Mucha; University of Cincinnati Medical Center -Kristin Hudock, Robert Duncan Hite*, Harshada More, Hammad Tanzeem, Carrie Keininger

PACIFIC NORTHWEST Clinical Center: Oregon Health and Science University - Catherine L. Hough*, Kelly Vranas, Akram Khan, Olivia F Krol, Madeline McDougal, Rupali Deshmukh, Zhengchun Luy, Milad Karami Jouzestani, Courtney Maxcy, Efrain Chavez Martinez, Otmar Borchard, Edlyn Lopez, Brennan Baylis; Harborview Medical Center -Nicholas J. Johnson, Bryce R.H. Robinson*, Stephanie Gundel, Sarah Katsandres, Megan R. Fuentes, Kelsey Jiang, Sarah G. Dean, Sakshi Sehgal

PITTSBURGH Clinical Center: University of Pittsburgh Presbyterian, Magee, Mercy, Shadyside -Donald M Yealy*, Alexandra Weissman, Sara Di Fore, Sarah McGarry, Tina Vita

© 2023 Admon AJ et al. *JAMA Network Open.*

SOUTHEAST Clinical Center: Wake Forest Baptist Health -Jessica Palakshappa, Chad Miller*, Clark Files*, Lori Flores, Mary LaRose, Rebecca Overman, Shannon Jones; University of Kentucky -Kirby Mayer, Peter Morris, Jamie Sturgill, Sara N Pasha, Evan Cassity; Medical University of South Carolina -Abbey Grady, Andy Goodwin

UTAH: Intermountain Medical Center -Samuel Brown*, Ithan Peltan, Joseph Bledsoe*, Jason Jacobs, Karah Imel, Brent Armbruster; Utah - LDS Hospital -Melissa Fergus, Valerie Aston; University of Utah Health Sciences Center -Estelle S Harris, Elizabeth A Middleton, Macy AG Barrios, Lindsey J Waddoups, Lindsey A McGuire

VANDERBILT Clinical Center: Vanderbilt University Medical Center -Todd W Rice*, Wesley H Self*, Margaret Hays, Sarah Frawley, Jakea Johnson; Duke University Medical Center -John Eppensteiner, Grace Hall, Alexander Limkakeng, Christopher Cox

Clinical Coordinating Center: Massachusetts General Hospital Biostatistics Center (CCC): B. Taylor Thompson*, Douglas L Hayden, Nancy Ringwood, Cathryn Oldmixon, Richard Morse, Ariela Muzikansky, Laura Fitzgerald, Samuel Whitaker, Haley Morin, Weixing Huang, Grace Carey

Johns Hopkins Medicine: Roy G Brower, Sarina Sahetya

National Heart, Lung, and Blood Institute: Lora Reineck, Neil Aggarwal, Karen Bienstock, Michelle Freemer, Myron Maclawiw, Gail Weinmann

Protocol Review Committee: Laurie J Morrison, Mark N Gillespie, Richard J Kryscio, Wojciech Zareba, Anne Rompalo, Michael Boeckh

Data and Safety Monitoring Board: Polly Parsons, Jason D Christie, Jesse R Hall, Nicholas J Horton, Laurie S Zoloth, Neal Dickert Jr., Deborah Diercks

**Clinical Center or CCC Principal Investigator*

© 2023 Admon AJ et al. *JAMA Network Open.*

Complete list of PETAL Network investigators

The National Heart, Lung, and Blood Institute Prevention and Early Treatment of Acute Lung Injury (PETAL) Clinical Trials Network:

Abi Fadel, Francois
Aboodi, Michael
Acquah, Samuel
Adams, Jason Y
Aggarwal, Neil
Alappan, Narendrakumar
Albertson, Timothy E
Al-Jaghbeer, Mohammed
Almasri, Eyad
Andrews, James S
Angus, Derek C
Baker, Jason V
Baron, Rebecca
Baumann, Michael
Becker, Torben
Biehl, Michelle
Bixby, Billie A
Bledsoe, Joseph R
Bloom, John W
Bose, Somnath
Boyle, Katherine L
Bradley, Patrick
Branson, Rich D
Brown, Samuel
Burnham, Ellen L
Calfee, Carolyn
Callahan, Sean J
Cancelliere, Alessandro
Carson, Shannon
Casey, Jonathan D
Chang, Steven Y
Chekuri, Sweta
Chen, Peter
Chen, Tina
Chenoweth, James
Choi, Philip
Co, Ivan
Collins, Sean
Costa Monteiro, Ana Carolina
Couper, Mick
Cox, Christopher
Daich, Jonathan
de Wit, Marjolein
deBoisblanc, Ben P
Dettmer, Matthew R
Dhar, Sanjay
Dillon, David
Doernberg, Sarah
Doshi, Pratik B
Douglas, Ivor S
Driver, Brian
Dugar, Siddharth P
Duggal, Abhijit
Elie, Marie-Carmelle
Enfield, Kyle B
Eppensteiner, John
Fein, Daniel
Filbin, Michael
Files, Clark
Finigan, James H.
Flannery, Alexander H.
Foulkes, Andrea
Fowler, Alpha A.
Frisch, Adam
Fung, Monica
Gaillard, John
Galbraith, James W
Gandotra, Sheetal
Gardner-Gray, Jayna M
Gay, Elizabeth A
Gibbs, Kevin
Gill, Evanpaul
Ginde, Adit
Girard, Timothy D
Go, Pauline H
Goel, Neha N
Goligher, Ewan C
Gomez-Arroyo, Jose
Gong, Michelle Ng
Goodwin, Andrew J
Gordon, Richard D
Grissom, Colin K
Gunnerson, Kyle
Han, Jin Ho
Hanane, Tarik
Hansen, Christopher K
Happel, Kyle
Harris, Estelle S
Haukoos, Jason
Hendey, Gregory W
Hendrickson, Carolyn M.
Hibbert, Kathryn A.
Hirshberg, Ellie
Hite, R. Duncan
Hope, Aluko A
Hoth, Jason
Hou, Peter C
Hough, Terri
Howrylak, Judie A
Huang, David T
Hudock, Kristin
Huebinger, Ryan
Hurst, Gina
Hypes, Cameron D
Hyzy, Robert C.
Iovine, Nicole
Islam, Marjan
Israel, Jennifer
Iwashyna, Jack
Jaffer, Faraz
Janssen, William
Jayaprakash, Namita
Jia, Shijing
Johnson, Nicholas J
Jolley, Sarah
Jones, Alan
Jones, Elizabeth Jones B
Kadl, Alex
Kangelaris, Kirsten N
Kashiouris, Markos G
Kass, Lawrence E
Keller, Marla
Khan, Akram
Khosravi, Mehdi
Khouli, Hassan
Kinni, Harish
Kiyatkin, Michael E
Knox, Daniel
Krishnan, Amita
Kurtzman, Nicholas
Lammi, Matthew R
Lanspa, Michael J
Le, Viet
Leither, Lindsay M
Levitt, Joseph
L'Heureux, Michael
Lim, George
Limkakeng, Alexander
Lin, Michael Y
Lindsell, Christopher
Liu, Kathleen
Lyn-Kew, Kenneth
Lyons, Patrick G
Mackay, Fraser C
Maher, Patrick J
Mahler, Simon A.
Malhotra, Anita

© 2023 Admon AJ et al. *JAMA Network Open.*

Malley, Brian E
Matthay, Michael A
May, Teresa
Mayer, Katherine
Mayer, Kirby P
McSparron, Jakob I
McVerry, Bryan J.
Miller, Chadwick D
Miller, Stephen
Minear, Steven C
Mireles, Eduardo
Mock, Jason
Mohamed, Amira
Montgomery-Yates, Ashley A
Morris, Peter
Mosier, Jarrod M
Moskowitz, Ari
Moss, Marc
Mucha, Simon R
Munroe, Elizabeth
Nair , Rahul
Nandi, Utsav
Nassisi, Denise
Nkemdirim, William
Norton, Dustin L
O'Gara, Brian
O'Hearn, Daniel J
OMahony, Darragh Shane
Page, David B
Paine, Robert
Park, Pauline K
Pasha, Sara
Patarroyo Aponte, Gabriel D
Patel, Avignat
Patel, Bela
Peltan, Ithan D.
Perman, Sarah
Pflaum-Carlson, Jacqueline Marie
Phang-Lyn, Simone
Pinto-Plata, Victor
Prekker, Matthew E
Puskarich, Mike
Qadir, Nida
Reddy, Raju M
Rice, Todd W
Richardson, Lynne D.
Riker, Richard R
Rivers, Emanuel P
Robinson, Bryce RH
Rogers, Angela J
Russell, Derek W.
Sahoo, Debasis
Sandrock, Christian
Sanne, Shane
Scheraga, Rachel G
Schmidt, Eric
Schooler, Jordan B
Schwab, Kristin
Self, Wesley H.
Semler, Matthew W
Sergot, Paulina
Shah, Faraaz
Shapiro, Nate
Shaw, Daniel L
Sherwin, Robert
Siuba, Matthew
Sjoding, Michael
Smithline, Howard
Sochor, Mark R
Spurzem, John R
Srinivasan, Vasisht
Steel, Tessa L
Steingrub, Jay S
Stenson, Bryan
Sterling, Sarah A
Stopyra, Jason
Stubblefield, William-May B.
Sturek, Jeffrey M
Sturgill, Jamie L
Swiderek, Jennifer
Syed, Aamer
Talmor, Daniel
Tatem, Geneva
Thiruchelvam, Nirosshan
Thompson, Taylor
Thompson Bastin, Melissa L
Tidswell, Mark
Torbati, Sam S
Trent, Stacy A
Trott, Terren
Van der Kloot, Thomas E
Vranas, Kelly C
Wang, Bonnie
Wang, Ralph
Ware, Lorraine
Waters, Christopher M.
Weissman, Alexandra J
Wick, Katherine D
Wilkin, Aimee M
Wilson, David M
Wilson, Jenny G
Wyles, David L
Yanta, Joseph H
Yealy, Donald M
Youngquist, Scott
Zaman, Tanzira

© 2023 Admon AJ et al. *JAMA Network Open.*
