# Supplementary material for: Assessment of Symptom, Disability, and Financial Trajectories in Patients Hospitalized for COVID-19 at 6 Months
Source: JAMA Netw Open. 2023 Feb 14;6(2):e2255795. doi: 10.1001/jamanetworkopen.2022.55795 (PMC9929698; doi:10.1001/jamanetworkopen.2022.55795)
Supplement: Supplement 2. — Nonauthor Collaborators. The National Heart, Lung, and Blood Institute PETAL Network [file jamanetwopen-e2255795-s002.pdf]

*Group Name(s): The National Heart, Lung, and Blood Institute Prevention and Early Treatment of Acute Lung Injury (PETAL) Network							
***First Name and Middle Initial(s)**	***Last Name**	***Suffix (eg, Jr, III)**	Academic Degrees	Institution	Location (city, state/province, country)	Role or Contribution, eg, chair, principal investigator	Group (if more than 1 Group listed in the byline) and/or Subgroup (eg, Steering Committee)
Francois	Abi Fadel						
Michael	Aboodi						
Samuel	Acquah						
Jason Y	Adams						
Neil	Aggarwal						
Narendrakumar	Alappan						
Timothy E	Albertson						
Mohammed	Al-Jaghbeer						
Eyad	Almasri						
James S	Andrews						
Derek C	Angus						
Jason V	Baker						
Rebecca	Baron						
Michael	Baumann						
Torben	Becker						
Michelle	Biehl						
Billie A	Bixby						
Joseph R	Bledsoe						
John W	Bloom						
Somnath	Bose						
Katherine L	Boyle						
Patrick	Bradley						
Rich D	Branson						
Samuel	Brown						
Ellen L	Burnham						
Carolyn	Calfee						
Sean J	Callahan						
Alessandro	Cancelliere						
Shannon	Carson						
Jonathan D	Casey						
Steven Y	Chang						
Sweta	Chekuri						
Peter	Chen						
Tina	Chen						
James	Chenoweth						
Philip	Choi						
Ivan	Co						
Sean	Collins						
Ana Carolina	Costa Monteiro						
Mick	Couper						
Christopher	Cox						

*First name, last name, and suffix (if applicable) are required and will appear in PubMed.

***First Name and Middle Initial(s)**	***Last Name**	***Suffix (eg, Jr, III)**	Academic Degrees	Institution	Location (city, state/province, country)	Role or Contribution, eg, chair, principal investigator	Group (if more than 1 Group listed in the byline) and/or Subgroup (eg, Steering Committee)
Jonathan	Daich						
Marjolein	de Wit						
Ben P	deBoisblanc						
Matthew R	Dettmer						
Sanjay	Dhar						
David	Dillon						
Sarah	Doernberg						
Pratik B	Doshi						
Ivor S	Douglas						
Brian	Driver						
Siddharth P	Dugar						
Abhijit	Duggal						
Marie-Carmelle	Elie						
Kyle B	Enfield						
John	Eppensteiner						
Daniel	Fein						
Michael	Filbin						
Clark	Files						
James H	Finigan						
Alexander H	Flannery						
Andrea	Foulkes						
Alpha A	Fowler						
Adam	Frisch						
Monica	Fung						
John	Gaillard						
James W	Galbraith						
Sheetal	Gandotra						
Jayna M	Gardner-Gray						
Elizabeth A	Gay						
Kevin	Gibbs						
Evanpaul	Gill						
Adit	Ginde						
Timothy D	Girard						
Pauline H	Go						
Neha N	Goel						
Ewan C	Goligher						
Jose	Gomez-Arroyo						
Michelle Ng	Gong						
Andrew J	Goodwin						
Richard D	Gordon						
Colin K	Grissom						
Kyle	Gunnerson						
Jin Ho	Han						

Supplemental Online Content: Nonauthor Collaborators
*First name, last name, and suffix (if applicable) are required and will appear in PubMed.

*First Name and Middle Initial(s)	*Last Name	*Suffix (eg, Jr, III)	Academic Degrees	Institution	Location (city, state/province, country)	Role or Contribution, eg, chair, principal investigator	Group (if more than 1 Group listed in the byline) and/or Subgroup (eg, Steering Committee)
Tarik	Hanane						
Christopher K	Hansen						
Kyle	Happel						
Estelle S	Harris						
Jason	Haukoos						
Gregory W	Hendey						
Carolyn M	Hendrickson						
Kathryn A	Hibbert						
Ellie	Hirshberg						
R. Duncan	Hite						
Aluko A	Hope						
Jason	Hoth						
Peter C	Hou						
Terri	Hough						
Judie A	Howrylak						
David T	Huang						
Kristin	Hudock						
Ryan	Huebinger						
Gina	Hurst						
Cameron D	Hypes						
Robert C	Hyzy						
Nicole	Iovine						
Marjan	Islam						
Jennifer	Israel						
Jack	Iwashyna						
Faraz	Jaffer						
William	Janssen						
Namita	Jayaprakash						
Shijing	Jia						
Nicholas J	Johnson						
Sarah	Jolley						
Alan	Jones						
Elizabeth Jones B	Jones						
Alex	Kadl						
Kirsten N	Kangelaris						
Markos G	Kashiouris						
Lawrence E	Kass						
Marla	Keller						
Akram	Khan						
Mehdi	Khosravi						
Hassan	Khouli						
Harish	Kinni						
Michael E	Kiyatkin						

*First name, last name, and suffix (if applicable) are required and will appear in PubMed.

*First Name and Middle Initial(s)	*Last Name	*Suffix (eg, Jr, III)	Academic Degrees	Institution	Location (city, state/province, country)	Role or Contribution, eg, chair, principal investigator	Group (if more than 1 Group listed in the byline) and/or Subgroup (eg, Steering Committee)
Daniel	Knox						
Amita	Krishnan						
Nicholas	Kurtzman						
Matthew R	Lammi						
Michael J	Lanspa						
Viet	Le						
Lindsay M	Leither						
Joseph	Levitt						
Michael	L'Heureux						
George	Lim						
Alexander	Limkakeng						
Michael Y	Lin						
Christopher	Lindsell						
Kathleen	Liu						
Kenneth	Lyn-Kew						
Patrick G	Lyons						
Fraser C	Mackay						
Patrick J	Maher						
Simon A	Mahler						
Anita	Malhotra						
Brian E	Malley						
Michael A	Matthay						
Teresa	May						
Katherine	Mayer						
Kirby P	Mayer						
Jakob I	McSparron						
Bryan J	McVerry						
Chadwick D	Miller						
Stephen	Miller						
Steven C	Minear						
Eduardo	Mireles						
Jason	Mock						
Amira	Mohamed						
Ashley A	Montgomery-Yates						
Peter	Morris						
Jarrod M	Mosier						
Ari	Moskowitz						
Marc	Moss						
Simon R	Mucha						
Elizabeth	Munroe						
Rahul	Nair						
Utsav	Nandi						
Denise	Nassisi						

*First name, last name, and suffix (if applicable) are required and will appear in PubMed.

***First Name and Middle Initial(s)**	***Last Name**	***Suffix (eg, Jr, III)**	Academic Degrees	Institution	Location (city, state/province, country)	Role or Contribution, eg, chair, principal investigator	Group (if more than 1 Group listed in the byline) and/or Subgroup (eg, Steering Committee)
William	Nkemdirim						
Dustin L	Norton						
Brian	O'Gara						
Daniel J	O'Hearn						
Darragh Shane	OMahony						
David B	Page						
Robert	Paine						
Pauline K	Park						
Sara	Pasha						
Gabriel D	Patarroyo Aponte						
Avignat	Patel						
Bela	Patel						
Ithan D	Peltan						
Sarah	Perman						
Jacqueline Marie	Pflaum-Carlson						
Simone	Phang-Lyn						
Victor	Pinto-Plata						
Matthew E	Prekker						
Mike	Puskarich						
Nida	Qadir						
Raju M	Reddy						
Todd W	Rice						
Lynne D	Richardson						
Richard R	Riker						
Emanuel P	Rivers						
Bryce RH	Robinson						
Angela J	Rogers						
Derek W	Russell						
Debasis	Sahoo						
Christian	Sandrock						
Shane	Sanne						
Rachel G	Scheraga						
Eric	Schmidt						
Jordan B	Schooler						
Kristin	Schwab						
Wesley H	Self						
Matthew W	Semler						
Paulina	Sergot						
Faraaz	Shah						
Nate	Shapiro						
Daniel L	Shaw						
Robert	Sherwin						
Matthew	Siuba						

Supplemental Online Content: Nonauthor Collaborators
*First name, last name, and suffix (if applicable) are required and will appear in PubMed.

*First Name and Middle Initial(s)	*Last Name	*Suffix (eg, Jr, III)	Academic Degrees	Institution	Location (city, state/province, country)	Role or Contribution, eg, chair, principal investigator	Group (if more than 1 Group listed in the byline) and/or Subgroup (eg, Steering Committee)
Michael	Sjoding						
Howard	Smithline						
Mark R	Sochor						
John R	Spurzem						
Vasisht	Srinivasan						
Tessa L	Steel						
Jay S	Steingrub						
Bryan	Stenson						
Sarah A	Sterling						
Jason	Stopyra						
William-May B	Stubblefield						
Jeffrey M	Sturek						
Jamie L	Sturgill						
Jennifer	Swiderek						
Aamer	Syed						
Daniel	Talmor						
Geneva	Tatem						
Nirosshan	Thiruchelvam						
Taylor	Thompson						
Melissa L	Thompson Bastin						
Mark	Tidswell						
Sam S	Torbati						
Stacy A	Trent						
Terren	Trott						
Thomas E	Van der Kloot						
Kelly C	Vranas						
Bonnie	Wang						
Ralph	Wang						
Lorraine	Ware						
Christopher M	Waters						
Alexandra J	Weissman						
Katherine D	Wick						
Aimee M	Wilkin						
David M	Wilson						
Jenny G	Wilson						
David L	Wyles						
Joseph H	Yanta						
Donald M	Yealy						
Scott	Youngquist						
Tanzira	Zaman						
