# Supplementary material for: Assessment of Symptom, Disability, and Financial Trajectories in Patients Hospitalized for COVID-19 at 6 Months
Source: JAMA Netw Open. 2023 Feb 14;6(2):e2255795. doi: 10.1001/jamanetworkopen.2022.55795 (PMC9929698; doi:10.1001/jamanetworkopen.2022.55795)
Supplement: Supplement 3. — Data Sharing Statement [file jamanetwopen-e2255795-s003.pdf]

Data Sharing Statement

Admon. Assessment of Symptom, Disability, and Financial Trajectories in Patients Hospitalized for COVID-19 at 6 Months. *JAMA Netw Open*. Published February 14, 2023. doi:10.1001/jamanetworkopen.2022.55795

Data
Data available: Yes
Data types: Deidentified participant data
How to access data: A deidentified repository of clinical, imaging, and biologic data and of biospecimens will be made available at the conclusion of the BLUE CORAL (estimated date below).
When available: beginning date: 01-01-2024

Supporting Documents
Document types: Statistical/analytic code
How to access documents: Statistical and analytical code will be made available by emailing ajadmon@umich.edu.
When available: With publication

Additional Information
Who can access the data: Deidentified data will be made available to researchers complying with requirements of the BLUE CORAL data sharing plan, approved by NHLBI and consistent with NIH data sharing policies.
Types of analyses: Scientific analyses
Mechanisms of data availability: Data will be made available after approval of a proposal with a signed data access agreement in accordance with NIH-funded research data sharing policies.
